# Visit probability and accessibility within space–time prism of activity program

**DOI:** 10.1080/13658816.2024.2378066

**Published:** 2024-07-17

**Authors:** Jing Lyu, Feixiong Liao, Soora Rasouli

**Affiliations:** Urban Planning and Transportation Group, Eindhoven University of Technology, Eindhoven, The Netherlands

**Keywords:** Accessibility, mobility trajectories, multi-state supernetwork, space–time prism, visit probability

## Abstract

Space–time prism (STP) is widely applied to measure individuals’ ability to reach opportunities given the resource limitations. The majority of STP models treat prism-based accessibility as binary measures, wherein all locations within a prism are assumed equally accessible while others are deemed inaccessible. Although a few STP models examined heterogeneous interiors by modeling visit probabilities within the STPs, they primarily focused on trip-level analysis and did not explore the application for accessibility measurement. This study proposes a model framework based on multi-state supernetworks for constructing and estimating the probabilistic STPs of daily activity programs. The estimation is implemented with latent class models to account for individual heterogeneities in travel and activity participation. Based on the probabilistic STPs, we suggest a space–time accessibility measurement incorporating visit probabilities. We validate the model framework using mobility trajectory data collected in the Netherlands and demonstrate that the visit probability model can effectively capture the probabilistic STP interiors and the proposed accessibility measurement can provide a comprehensive evaluation of accessibility in the presence of activity chains.

## 1. Introduction

Individuals’ potential mobility is subject to space–time constraints given resource limitations. The space–time prism (STP) is a central time geographic concept that delimits the space–time opportunities that can be reached by a moving object (Hägerstrand 1970, Kuijpers and Technitis 2020). The classic STP is determined by the known anchor points, time budget, the maximum attainable travel speed, and the time available for a flexible activity that can be conducted at one of multiple activity locations (Lenntorp 1977). An STP envelopes all possible space–time paths and contains the potential path area (PPA) projected onto the 2-D planar space. The STP over a transportation network, also referred to as network-time prism (NTP), is established to delimit the accessible locations with respect to geometries, connectivity, and the time constraints imposed by the spatial network (Miller 1991). STP and PPA have been widely used to measure space–time accessibility (Miller 2017, Qin and Liao 2024). The majority of STP models treat prism-based accessibility as binary measures such that all locations within the prisms are considered equally accessible with deterministic characterizations, otherwise not accessible.

In reality, the probability of an individual visiting locations within reachable areas is not equally distributed. In that sense, an STP does not have homogeneous interiors. To transform the STP from the binary measurement to the probabilistic measurement, the visit probability distribution within a prism has been modeled. Specifically, to capture the variations within a prism, Winter (2009) presented the first steps into the probabilistic time geography by quantifying the probabilities of an individual visiting potential locations at a moment in time. The probability of an individual being at certain locations at sequential time steps is calculated by applying a convolution kernel on the units of 2-D coordinate recursively. Winter and Yin (2010a) simulated the probability distribution within a planar STP at each discrete time using unbiased random walks without a direction. Considering that an individual behaves with goal-orientation, Winter and Yin (2010b) further introduced the direction from an origin to a destination applying the bivariate normal distribution as a function of time. Song and Miller (2014) extended their work by formulating direct random walk (DRW) and truncated Brownian bridge (TBB) as discrete and continuous stochastic models for simulating the visit probabilities. In Song *et al*. (2016), to explore the probabilities of movements constrained by the transportation network, the visit probability within an STP is modeled based on a continuous-time semi-Markov process. The results of the simulation showed that the visit probability provides an appropriate quantitative description of individuals’ potential mobility. These studies modeled STPs predominantly at the trip level for a single flexible activity, referred to as trip-based STPs from the perspective of travel demand modeling, rather than for a daily activity program (AP) of multiple activities that potentially involve complex activity chains and larger spatial and temporal coverages.

Relatively, only a few studies extended the standard trip-based STPs to the so-called activity-based STPs that aim to capture the interdependencies between multiple activities. To accommodate flexible APs, the existing STP models are applied in the presence of activity chains by sequentially constructing the trip-based STPs given pre-defined activity sequences as building STP blocks (Kuijpers 2017), which underestimates the real STP by disregarding the contributions from other activity sequences. On the other hand, Kang and Chen (2016) constructed the feasible space–time region for a daily AP by intersecting feasible space–time regions of all single activities, which overestimates the real STP since the feasible space–time regions of single activities are exaggerated when accumulating durations of multiple activities. To address these limitations, Liao (2021) proposed bidirectional searches of full activity-travel patterns (ATPs) through multi-state supernetworks that can delineate the exact STP for an AP efficiently (see page 2 in the Supplementary document for the illustration of trip-based and activity-based STPs). It should be noted that all these STP model extensions apply binary measurement.

Therefore, we argue that it is essential to construct the probabilistic STP of an individual’s AP considering activity chains. First, constructing the probabilistic STP of an AP as opposed to a single trip is of value since trip chaining represents a higher realism of daily mobility. Second, using non-uniform visit probabilities, which reflect how likely the locations within the STP can be visited at a moment in time, have higher realism than the deterministic counterparts. Third, different from a trip-based STP involving only one flexible activity, a daily AP usually involves multiple activities, which tend to cause irregular geometrical structures at different activity states due to the flexible activity sequences and locational time window constraints. As a result, it is not straightforward to determine the visit probabilities simply by checking whether the locations are geographically near the shortest path between the anchor points.

By delimiting all space–time points that can be reached by an individual given limited resources, the STP is the basis for conceptualizing and operationalizing the people-based space–time accessibility measurement. Unlike place-based accessibility measures based on the concept of proximity, the space–time accessibility measures have the advantage of interconnecting an individual’s activity schedule and the urban system (Lenntorp 1978). The number of opportunities in the PPA is usually used as an indicator, reflecting the action space for an individual to participate in activities. Notably, Neutens *et al*. (2010) compiled six people-based measures, which were widely applied in different contexts to evaluate accessibility to various services and compared their application in producing equity measures (e.g. Ren *et al*. 2014, Lee and Miller 2019, Qin and Liao 2021). Similarly, all these measures treat the opportunities inside the STP as binarily accessible, ignoring the probabilistic characterization in space and time.

In view of the above, this study aims to develop a model framework for twofold purposes: (1) to propose a method to construct and estimate the probabilistic STP of conducting a daily AP with flexible activity chains, and (2) to suggest a space–time accessibility measurement based on the probabilistic STP. The method of constructing the activity-based probabilistic STP includes three steps. First, we construct the exact STP for a daily AP based on the multi-state supernetwork representation (Liao 2019, 2021). Second, we model the visit probability within the STP using semi-Markov techniques (Song *et al*. 2016). Considering the heterogeneity across individuals, we use latent class models to formulate the holding time density functions for travel and activity participation to model the heterogeneous extra time on the links of the supernetwork. Third, we estimate the visit probability model using mobility trajectories collected in the Netherlands based on the method of maximum likelihood estimation (MLE). Based on the probabilistic STP, we demonstrate a measurement incorporating the probabilistic STP to evaluate space–time accessibility considering heterogeneous STP interiors.

The remainder of this paper is organized as follows. Section 2 constructs the activity-based probabilistic STP, discusses the estimation of the visit probability model, and proposes the accessibility measurement incorporating visit probability. Section 3 validates the proposed methods using mobility trajectory data. The paper is completed with conclusions and plans for future work in Section 4.

The following abbreviations are used in this study.

**Table:** 

STP:	Space–time prism	AP:	Activity program
PPA:	Potential path area	SMP:	Semi-Markov process
NTP:	Network-time prism	*NAL*:	Number of accessible locations
*SNK*:	Multi-state supernetwork	*AFT*:	Aggregate flexible time
TBS:	Two-stage bidirectional search	*MFT*:	Maximum flexible time

## 2. Modeling

This section first delineates the exact STP of an AP in a multi-state supernetwork. Based on the activity-based STP, the visit probabilities of the links in the supernetwork are modeled using the method of semi-Markov process (SMP). Next, heterogeneous link holding time density functions are estimated with extracted mobility trajectories. Finally, an accessibility measurement is proposed.

### 2.1. Activity-based STP and heterogeneous holding time density functions

**Figure 1.**
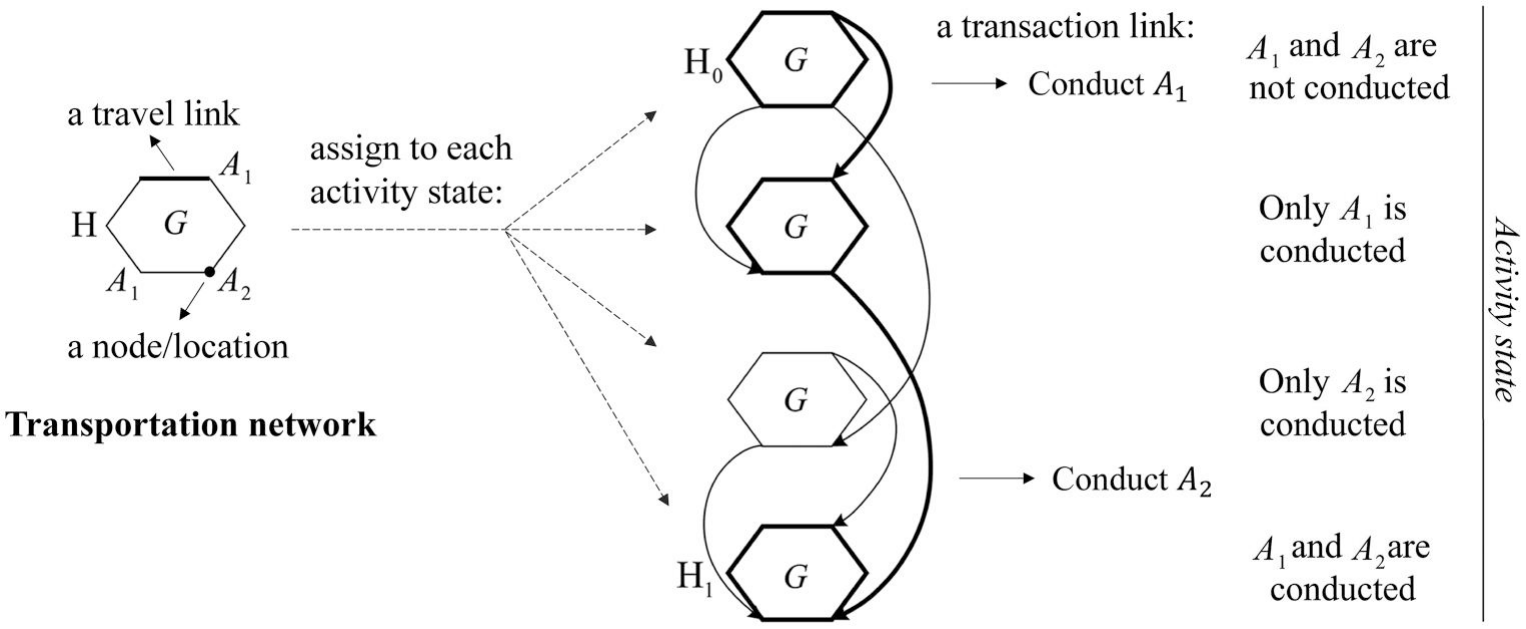
Multi-state supernetwork representation with a single mode.

Multi-state supernetworks are capable of representing the ATP space for conducting an individual’s AP (Liao *et al*. 2010, 2013). To distinguish different stages of the implementation of an AP, we assign a copy of the transportation network to each possible activity state, specifying which activities have been conducted. The link that interconnects the same activity location at two different reachable activity states is considered an ‘activity link’ or ‘transaction link’. An activity link leads to a change in activity states, thereby representing the participation of an activity at a location. If there are multiple locations for an activity, there are as many activity links as locations for one episode of activity state changes. In such a way, a daily AP’s implementation is a path choice through a network of networks of different activity states. Using a hexagon G to denote a unimodal transportation network and the vertices of G to denote representative intersections and activity locations, a multi-state supernetwork with a single transportation mode for conducting two activities is illustrated in Figure 1 (undirected travel links in G are bi-directed), which includes four activity states due to the flexible activity sequences. As shown, activity $A_{1}$ has two location alternatives and $A_{2}$ only has one, which results in two activity links for conducting $A_{1}$ and a single activity link for $A_{2}$ for one episode of activity state changes. The ATP expressed by the interconnected bold links indicates that an individual leaves home ($H_{0}$) and conducts activity $A_{1}$ at one of the two locations and then conducts activity $A_{2}$ before returning home ($H_{1}$), where origin $H_{0}$ is at the first activity state and destination $H_{1}$ is at the last activity state (see page 4 in the Supplementary document for illustrating different activity sequences). Denote the multi-state supernetwork as SNK(N,E), where node set N includes road intersections, activity locations, and parking locations, and link set E includes travel links of road segments and transaction links for conducting activities at activity locations.

Suppose $H_{0}$ and $H_{1}$ are two anchors with the corresponding time budget window $[t_{H_{0}},t_{H_{1}}].$ An individual conducts activity α from an activity set A at one of multiple locations with a minimum duration $d_{\alpha }.$ The condition that node n of G falls within the STP and PPA in the planar space is delimited by

(1) $$L(H_{0},n)+\sum_{\alpha \in A}d_{\alpha }+L(n,H_{1})\leq t_{H_{1}}-t_{H_{0}}\;$$

where L(∙,∙) denotes the travel time derived by the Euclidean distance between two nodes and the maximum travel speed in the transportation network. Equation (1) describes the upper bound STP and PPA. Considering STP and PPA over a real transportation network, the temporal feasibility for a node in *SNK* in the STP and PPA is formulated as

(2) $$\text{min}\;\left\{g(H_{0},{n|}_{s})+g({n|}_{s},H_{1})\right\}\leq t_{H_{1}}-t_{H_{0}}$$

where ${n|}_{s}$ denote node n at activity state s in *SNK*, $g(H_{0},{n|}_{s})$ and $g({n|}_{s},H_{1})$ are the actual activity-travel times of two sub-paths from $H_{0}$ to ${n|}_{s}$ and ${n|}_{s}$ to $H_{1},$ respectively. The two-stage bidirectional search (TBS) methods (Liao 2021) can be applied to find the exact STP of an AP. The TBS algorithm first generates a search space by performing a forward search from $H_{0}$ using $\text{min}\;\left\{g(H_{0},{n|}_{s})+h({n|}_{s},H_{1})\right\}$ as the labeling criterion, where $h({n|}_{s},H_{1})$ is an estimation of $g({n|}_{s},H_{1}),$ then performs the backward search in the reversed *SNK* from $H_{1}$ in the generated forward search space to find the real PPA. The STP comprises all ${n|}_{s}\in \text{SNK}$ that satisfy Equation (2) at time range $\left[t_{H_{0}}+\text{min}\;g(H_{0},{n|}_{s}),t_{H_{1}}-\text{min}\;g({n|}_{s},H_{1})\right]$ for an AP.

Based on the constructed activity-based STP in *SNK*, the visit probabilities within the STP can be modeled using SMP, which requires a function that describes the probability for the process to hold a specific amount of time before transitioning from one state to another. Holding the principles of SMP unchanged, we redefine the state in SMP as a ‘status’ to avoid conflicting definitions with the ‘state’ in *SNK*. Status is defined as a movement starting from node ${i|}_{s}$ to node ${j|}_{s^{\prime }}$ along link $l_{\text{ij}|ss^{\prime }}$ in *SNK* but has not arrived at ${j|}_{s^{\prime }}$ yet, $l_{\text{ij}|ss^{\prime }}=({i|}_{s},{j|}_{s^{\prime }})$ for $\forall s,s^{\prime }.$ The status space includes the movements on all possible links within the STP. Since both travel and activity participation are represented as links, there is no difference in the definitions of ‘status’ for links in *SNK*. Specifically, the possible movements on an activity link may be either proceeding to complete the activity or quitting the activity before it is completed. As ‘moving along a link’ for travel and activity are independent, we assume uniform status transition probabilities along each link in *SNK*.

Song *et al*. (2016) defined the homogeneous holding time density function to describe the probability of moving on a road segment. To extend the model for an AP, we modify the holding time density function to describe the probability that the transition from node ${i|}_{s}$ to ${j|}_{s^{\prime }}$ will take an extra time τ beyond the minimum time expense on $l_{\text{ij}|ss^{\prime }}.$ For the sake of simplicity, we define $e=l_{\text{ij}|ss^{\prime }}.$ The holding time density function is denoted by $f_{e}(\tau )$ when τ≥0, otherwise equals 0. The extra time τ on link e can be calculated using $\tau =Τ-t_{e}^{\text{min}},$ where Τ is the total time traversing link e and $t_{e}^{\text{min}}$ is the minimum time expense on e. $f_{e}(\tau )$ can also be interpreted as the probability of reaching ${j|}_{s^{\prime }}$ with arrival time $t_{e}^{\text{min}}$ + τ. For a travel link, i≠j and $s=s^{\prime },$ $t_{e}^{\text{min}}$ is the minimum travel time; for a transaction link, i=j and $s{\neq s}^{\prime },$ $t_{e}^{\text{min}}$ corresponds to the minimum activity duration $d_{\alpha }.$

Considering that the individuals may behave differently on travel and different activity participation, we consider heterogeneous $f_{e}(\tau )$ for travel and transaction links. We apply a latent class model to capture discrete heterogenous holding time functions using individuals’ socio-demographic characteristics in the membership functions (Hensher *et al*. 2015). Given K latent classes, the probability that an individual q belongs to latent class k is formulated as

(3) $$P_{\text{qk}}=\frac{\text{exp}\;\left(\beta_{k}x_{q}\right)}{\sum_{k^{\prime }=1}^{K}\text{exp}\;\left(\beta_{k^{\prime }}x_{q}\right)},k,k^{\prime }=1,\ldots ,K,\beta_{K}=0$$

where $x_{q}$ is a vector of socio-demographic characteristics of q, $\beta_{k}$ is a parameter vector of $x_{q}$ of latent class k. The K-th parameter vector is fixed to zero to secure model identification. The latent class holding time density function in *SNK* is formulated as

(4) $$f_{e}^{q}(\tau )=\sum_{k=1}^{K}P_{\text{qk}}∙f_{e}^{q}(\tau |k),e\in \text{SNK}$$

where $f_{e}^{q}(\tau |k)$ denotes the probability of individual q in class k for spending extra time τ on link e in *SNK*, and $f_{e}^{q}(\tau )$ is the probability of holding time τ of individual q on e. The same population group classified into one latent class would have the same estimations of holding time density functions towards different types of links.

### 2.2. Estimation of holding time density functions

To estimate the heterogeneous holding time density functions, we first need to set the extra available times on travel and activity participation from the observed mobility trajectories. One trajectory records the daily ATP of an individual by a sequence of GPS positions, and travel time and activity duration can be inferred from the timestamps associated with GPS coordinates.

For travel links, the extra time of a sampled individual ATP is calculated as

(5) $$\tau =\text{rec}(t_{\text{ATP}})-\text{min}\;\left(t_{\text{ATP}}\right)$$

where $\text{rec}(t_{\text{ATP}})$ is the recorded ATP time obtained from the mobility trajectory, and $\text{min}\;\left(t_{\text{ATP}}\right)$ is the shortest ATP time given the same activity durations in $\text{rec}(t_{\text{ATP}}),$ which can be found using the standard shortest path searching algorithm in *SNK*. Therefore, the extra travel time is obtained by the subtraction of the two values. Typically, the longer ‘extra time’ for travel, the less likely an individual would remain on travel links. As an extension in the latent class domain and based on the empirical distribution of the GPS trajectories, the form of holding time density function $f_{e}^{q}(\tau |k)$ of travel link e can be described by an exponential distribution as

(6) $$f_{e}^{q}(\tau |\lambda_{k})=\{\begin{array}{cc} \lambda_{k}e^{-\lambda_{k}\tau }, & \tau \geq 0\\ 0, & \tau <0 \end{array}$$

In such a way, $f_{e}^{q}(\tau |k)$ in Equation (4) is substituted by Equation (6) as $f_{e}^{q}(\tau |\lambda_{k})$ with parameter $\lambda_{k}$ to be estimated. For transaction links, the extra activity duration of a sampled individual ATP is calculated as

(7) $$\tau_{\alpha }=\text{rec}(d_{\alpha })-\text{min}\;\left(d_{\alpha }\right)$$

where $\text{rec}(d_{\alpha })$ is the recorded duration for conducting activity α, and $\text{min}\;\left(d_{\alpha }\right)$ is the minimum duration of activity α among all the extracted ATPs. Individuals expect to finish the activity with an ideal duration, which is usually longer than the minimum activity duration and less likely to be overlong. With this principle and also based on the empirical distribution of the GPS trajectories, the form of holding time density function $f_{e}^{q}(\tau |k)$ for conducting an activity can be described by a lognormal distribution as

(8) $$f_{e}^{q}(\tau |\mu_{k},\sigma_{k}^{2})=\frac{1}{\tau \sigma_{k}\sqrt{2\pi }}\text{exp}\;\left(-\frac{{\left(\text{ln}\;\left(\tau \right)-\mu_{k}\right)}^{2}}{2\sigma_{k}^{2}}\right),\tau >0$$

$f_{e}^{q}(\tau |k)$ in Equation (4) is substituted by Equation (8) as $f_{e}^{q}(\tau |\mu_{k},\sigma_{k}^{2})$ with parameters $\mu_{k}$ and $\sigma_{k}\;(k=1,2,\ldots ,K)$ to be estimated. $f_{e}^{q}(\tau |\mu_{k},\sigma_{k}^{2})=0$ if τ=0.

Based on Equations (6) and (8), we can estimate the parameters using the method of MLE. Given Q ATP trajectories, the likelihood for the observations from a sample is given by

(9) $$L=\prod_{q=1}^{Q}\left[\sum_{k=1}^{K}P_{\text{qk}}∙f_{e}^{q}(\tau |k)\right]=\prod_{q=1}^{Q}\left[\sum_{k=1}^{K}\frac{\text{exp}\;\left(\beta_{k}x_{q}\right)}{\sum_{k^{\prime }=1}^{K}\text{exp}\;\left(\beta_{k^{\prime }}x_{q}\right)}∙f_{e}^{q}(\tau |\theta_{k})\right]$$

where $\theta_{k}$ is a vector of parameters in Equations (6) and (8). The log-likelihood for the sampled mobility trajectories is:

(10) $$\text{ln}\;L=\sum_{q=1}^{Q}\left(\text{ln}\;\sum_{k=1}^{K}P_{\text{qk}}∙f_{e}^{q}(\tau |\theta_{k})\right)$$

The MLE of the proposed latent class models is implemented with the method of expectation–maximization (EM) algorithm. The estimated parameters are used to simulate the visit probabilities within the constructed activity-based STP. Since visit probabilities and the visit probability-based accessibility measurements are individual-specific in a latent class, the notations denoting individual q and latent class k in $f_{e}^{q}(\tau |k)$ are omitted below for the sake of conciseness.

### 2.3. Visit probability formulation

In the activity-based STP, the earliest arrival time $t_{{i|}_{s}}^{-}$ at node ${i|}_{s}$ and the latest departure time $t_{{j|}_{s^{\prime }}}^{+}$ from node ${j|}_{s^{\prime }}$ can be obtained for each link e in *SNK*. The visit probability in *SNK* as an extension over Song *et al*. (2016) is defined as follows.

Let $P_{H_{0}\to {i|}_{s}}(t)$ denote the probability that e can be reached via ${i|}_{s}$ at t from the origin $H_{0}.$ Given τ as the extra time available and $t_{e}^{\text{min}}$ as the minimum time expense on e, the range of t arriving at e starts from $t_{{i|}_{s}}^{-}$ and is not later than $t_{{j|}_{s^{\prime }}}^{+}-t_{e}^{\text{min}}.$ When $t\in [t_{{i|}_{s}}^{-},t_{{j|}_{s^{\prime }}}^{+}-t_{e}^{\text{min}})$, $P_{H_{0}\to {i|}_{s}}(t)$ is calculated as the probability of spending extra time τ on e by integrating $f_{e}(\tau )$ over interval $\left[t_{{i|}_{s}}^{-}-t_{H_{0}},t-t_{H_{0}}\right]$ given the reference departure time $t_{H_{0}}.$ Otherwise, $P_{H_{0}\to {i|}_{s}}(t)$ is 0 when t is earlier than $t_{{i|}_{s}}^{-},$ and a constant when t is later than $t_{{j|}_{s^{\prime }}}^{+}-t_{e}^{\text{min}}$ within the time budget $[t_{H_{0}},t_{H_{1}}].$ $P_{H_{0}\to {i|}_{s}}(t)$ is formulated as

(11) $$P_{H_{0}\to {i|}_{s}}(t)=\{\begin{array}{ll} 0\; & t\in [t_{H_{0}},t_{{i|}_{s}}^{-})\;\\ \int_{t_{{i|}_{s}}^{-}-t_{H_{0}}}^{t-t_{H_{0}}}f_{e}(\tau )d\tau \; & t\in [t_{{i|}_{s}}^{-},t_{{j|}_{s^{\prime }}}^{+}-t_{e}^{\text{min}})\\ \int_{t_{{i|}_{s}}^{-}-t_{H_{0}}}^{t_{{j|}_{s^{\prime }}}^{+}-t_{e}^{\text{min}}-t_{H_{0}}}f_{e}(\tau )d\tau \; & t\in [t_{{j|}_{s^{\prime }}}^{+}-t_{e}^{\text{min}},t_{H_{1}}] \end{array}$$

Similarly, let $P_{{j|}_{s^{\prime }}\to H_{1}}(t)$ denote the probability of reaching $H_{1}$ from ${j|}_{s^{\prime }}$ based on possible departure times. The range of t departing from e starts from $t_{{i|}_{s}}^{-}+t_{e}^{\text{min}}$ and is no later than $t_{{j|}_{s^{\prime }}}^{+}.$ Thus, when $t\in [t_{{i|}_{s}}^{-}+t_{e}^{\text{min}},t_{{j|}_{s^{\prime }}}^{+}),$ $P_{{j|}_{s^{\prime }}\to H_{1}}(t)$ can be calculated by integrating $f_{e}(\tau )$ over the interval $\left[t_{H_{1}}-t,t_{H_{1}}-t_{{j|}_{s^{\prime }}}^{+}\right]$ given the reference arrival time $t_{H_{1}}.$ $P_{{j|}_{s^{\prime }}\to H_{1}}(t)$ is 0 when t is later than $t_{{j|}_{s^{\prime }}}^{+}$ and a constant when t is earlier than $t_{{i|}_{s}}^{-}+t_{e}^{\text{min}}.$ $P_{{j|}_{s^{\prime }}\to H_{1}}(t)$ is formulated as

(12) $$P_{{j|}_{s^{\prime }}\to H_{1}}(t)=\{\begin{array}{ll} 0\; & t\in [t_{{j|}_{s^{\prime }}}^{+},t_{H_{1}}]\\ \int_{t_{H_{1}}-t_{{j|}_{s^{\prime }}}^{+}}^{t_{H_{1}}-t}f_{e}(\tau )d\tau \; & t\in [t_{{i|}_{s}}^{-}+t_{e}^{\text{min}},t_{{j|}_{s^{\prime }}}^{+})\\ \int_{t_{H_{1}}-t_{{j|}_{s^{\prime }}}^{+}}^{t_{H_{1}}-t_{{i|}_{s}}^{-}-t_{e}^{\text{min}}}f_{e}(\tau )d\tau \; & t\in [t_{H_{0}},t_{{i|}_{s}}^{-}+t_{e}^{\text{min}}) \end{array}$$

The probability of visiting link e at $t\in [t_{H_{0}},t_{H_{1}}],$ denoted by P(e,t), is formulated as the joint probability of e being reached from ${i|}_{s}$ and arriving at $H_{1}$ from ${j|}_{s^{\prime }}$ within $t_{H_{1}}-t.$ We normalize the probabilities among all accessible links in *SNK* at time t, with $\sum_{e\in E}P(e,t)=1$ over the status space at time t. After normalization, P(e,t) is formulated as

(13) $$P(e,t)=\{\begin{array}{cc} \frac{P_{H_{0}\to {i|}_{s}}(t)\times P_{{j|}_{s^{\prime }}\to H_{1}}(t)}{\sum_{e^{\prime }\in E}P(e^{\prime },t)}, & \text{if }t_{{i|}_{s}}^{-}+t_{e}^{\text{min}}\leq t_{{j|}_{s^{\prime }}}^{+}\\ 0, & \text{otherwise}\; \end{array}$$

Equation (13) can be used to derive the visit probability for each link in *SNK* at a time moment in the status space given the appropriate holding time density functions (see page 5 in the Supplementary document for illustrating the visit probability model in *SNK*). For conducting an out-of-home activity α at location i with duration $d_{\alpha },$ denote the time window constraint as $[o_{\alpha i},c_{\alpha i}],d_{\alpha }+o_{\alpha i}\leq c_{\alpha i},$ where $o_{\alpha i}$ and $c_{\alpha i}$ are the opening and closing times of location i respectively. Equations (11) and (12) can be modified to accommodate the time window constraints. For $t_{{i|}_{s}}^{-}$ of transaction link e, $t_{{i|}_{s}}^{-}=o_{\alpha i}$ if $t_{{i|}_{s}}^{-}<o_{\alpha i}$ and remains unchanged if $o_{\alpha i}\leq t_{{i|}_{s}}^{-}\leq c_{\alpha i};$ if $t_{{i|}_{s}}^{-}>c_{\alpha i},$ $P_{H_{0}\to {i|}_{s}}(t)$ is 0 since e cannot be reached within $[o_{\alpha i},c_{\alpha i}].$ Similarly, $t_{{j|}_{s^{\prime }}}^{+}=c_{\alpha i}$ if $t_{{j|}_{s^{\prime }}}^{+}>c_{\alpha i}$ and remains unchanged if $t_{{j|}_{s^{\prime }}}^{+}$ is within $[o_{\alpha i},c_{\alpha i}];$ if $t_{{j|}_{s^{\prime }}}^{+}<o_{\alpha i},$ $P_{{j|}_{s^{\prime }}\to H_{1}}(t)$ is 0 given the time window constraint. At last, if $t_{{i|}_{s}}^{-}+t_{e}^{\text{min}}>t_{{j|}_{s^{\prime }}}^{+},$ $f_{e}(\tau )=0$ since there is no extra time on e.

### 2.4. Space–time accessibility incorporating visit probability

Based on the deterministic STP, the number of accessible locations (*NAL*), aggregate flexible time (*AFT*), and maximum flexible time (*MFT*) are commonly applied accessibility measures (Neutens *et al*. 2010, Qin and Liao 2021). *NAL*, *AFT*, and *MFT* count the number of locations, the accumulated flexible time of all accessible locations, and the maximum duration that can be spent at one location, respectively. Let e represent a transaction link (or activity link) between activity states s and $s^{\prime }$ for conducting an activity at a location in *SNK* and $\tilde{s}$ denote the activity state transition $s\to s^{\prime }$ for short, *NAL*, *AFT*, and *MFT* can be measured for each $\tilde{s}$ with flexible activity sequences as

(14) $$\text{NAL}(\tilde{s})=\sum_{e}R(e),R(e)=\{\begin{array}{l} 1,\text{if }\text{e}:s\to s^{\prime }\in \text{STP}\\ 0,\text{otherwise}\; \end{array}\;$$

(15) $$\text{AFT}(\tilde{s})=\sum_{e}\left(t_{{j|}_{s^{\prime }}}^{+}-t_{{i|}_{s}}^{-}-d_{\alpha }^{\text{min}}\right)R(e),i=j$$

(16) $$\text{MFT}(\tilde{s})=\underset{e}{\text{max}}\left[\left(t_{{j|}_{s^{\prime }}}^{+}-t_{{i|}_{s}}^{-}-d_{\alpha }^{\text{min}})R(e\right)\right],i=j$$

where R(e) indicates if the associated activity α with the minimum activity duration $d_{\alpha }^{\text{min}}$ can be conducted via link e or not, delimited by Equation (2). Combining Equations (14)–(16), we propose a space–time accessibility measure that considers the heterogeneous STP interior incorporating Equation (13). For the capture of state-time dependency, we denote the set of all activity links between state s and $s^{\prime }$ in the STP at t as $\zeta (\tilde{s},t)=\{e|e\in \text{STP}\;\text{at}\;t\}.$

With $\zeta (\tilde{s},t),$ a more realistic space–time accessibility measurement $A(\tilde{s},t)$ is proposed to take into account the attractiveness and visit probability of the activity locations within the probabilistic STP. The generic accessibility measurement is formalized as

(17) $$A(\tilde{s},t)=\sum_{e}f(G(e,t),P(e,t)),e\in \zeta (\tilde{s},t)$$

where f is a function of the attractiveness G(e,t) and visit probability P(e,t) of activity link $e\in \zeta (\tilde{s},t).$ Unlike previous measures, Equation (17) accounts for the visit probability of each activity location at $\tilde{s}$ and t. The following remarks are made.

Remark 1: Keeping P(e,t),∀e unchanged, an increase in G(e,t) of any activity location in $\zeta (\tilde{s},t)$ leads to an increase in accessibility.

Remark 2: Keeping G(e,t),∀e unchanged, an increase in P(e,t) of an activity location in $\zeta (\tilde{s},t)$ does not necessarily increase accessibility. With Equation (13), the increase of P(e,t) of one location is associated with the decreased visit probabilities of other locations. When the attractiveness of such locations is even higher, the system accessibility may be worse off.

Remark 3: The addition of a location in $\zeta (\tilde{s},t)$ does not necessarily increase accessibility. Similarly, the addition of the location reduces the visit probabilities of existing locations. If those locations have high attractiveness, the system accessibility may be worse off. The proposed accessibility measure differs from the existing ones, of which the addition of locations always increases or at least does not reduce accessibility.

Remark 4: When there are variations in attractiveness, visit probability, and the number of accessible locations, a trade-off needs to be considered when evaluating the accessibility.

Given the discrete distribution of activity locations, the corresponding visit probabilities are also discrete, represented as $P=\{P(e_{i},t),i=1,\ldots ,|\zeta (\tilde{s},t)|\}$ based on Equation (13). The corresponding locations’ attractiveness is $G=\{G(e_{i},t),i=1,\ldots ,|\zeta (\tilde{s},t)|\}.$ To incorporate visit probabilities for refining accessibility measurement based on the traditional methods, we can first make a single-point transformation. For any $P(e_{i},t)\in (0,1),$ a general transformation can be formulated using a power law transformation function as

(18) $$\hat{P}(e_{i},t)={\left(P(e_{i},t)\right)}^{\gamma }$$

where $\hat{P}(e_{i},t)$ is the transformed visit probability from $P(e_{i},t)$ and γ is the transformation parameter. If 0≤γ<1, $\hat{P}(e_{i},t)$ overweighs $P(e_{i},t)$ since $\hat{P}(e_{i},t)>P(e_{i},t);$ if γ=1, $\hat{P}(e_{i},t)=P(e_{i},t);$ if γ>1, $\hat{P}(e_{i},t)$ underweighs $P(e_{i},t)$ since $\hat{P}(e_{i},t)<P(e_{i},t).$ The transformation integrates and extends the methods of traditional accessibility measures. For instance, *MFT* allows the accessibility of the location with the highest flexible time to greatly outweigh that of the others, while *AFT* assigns an equivalent weight to the flexible time of each accessible location. Similar to Equation (16), to highlight the contribution of the most likely visited location, $\hat{P}(e_{i},t)$ can be formulated as

(19) $$\hat{P}(e_{i},t)=\{\begin{array}{ll} 1, & \text{if }i=\text{arg}\underset{j}{\text{max}}\left\{P(e_{j},t),j\in \{1,\ldots ,|\zeta (\tilde{s},t)|\right\}\;\\ P(e_{i},t), & \text{otherwise}\; \end{array}\;$$

where i in the first condition refers to an activity location with the highest visit probability. In case there are multiple such activity locations, the delimiting condition should be extended to refer to the one with the highest attractiveness. Equation (19) is a special case of Equation (18) that integrates Equation (13) and the traditional measurements. The transformation describes that a location with the highest visit probability is overweighed (γ=0), while other locations are accessed with their original visit probability (γ=1). To account for the number of locations in Equation (14) and the accumulated attractiveness (flexible duration is one example of attractiveness) in Equation (15), a realization of Equation (17), representing the aggregate attractiveness with visit probability (*AVP*), is formulated as

(20) $$\text{AVP}(\tilde{s},t)=\sum_{i=1}^{\left|\zeta (\tilde{s},t)\right|}G(e_{i},t)∙\hat{P}(e_{i},t)$$

**Figure 2.**
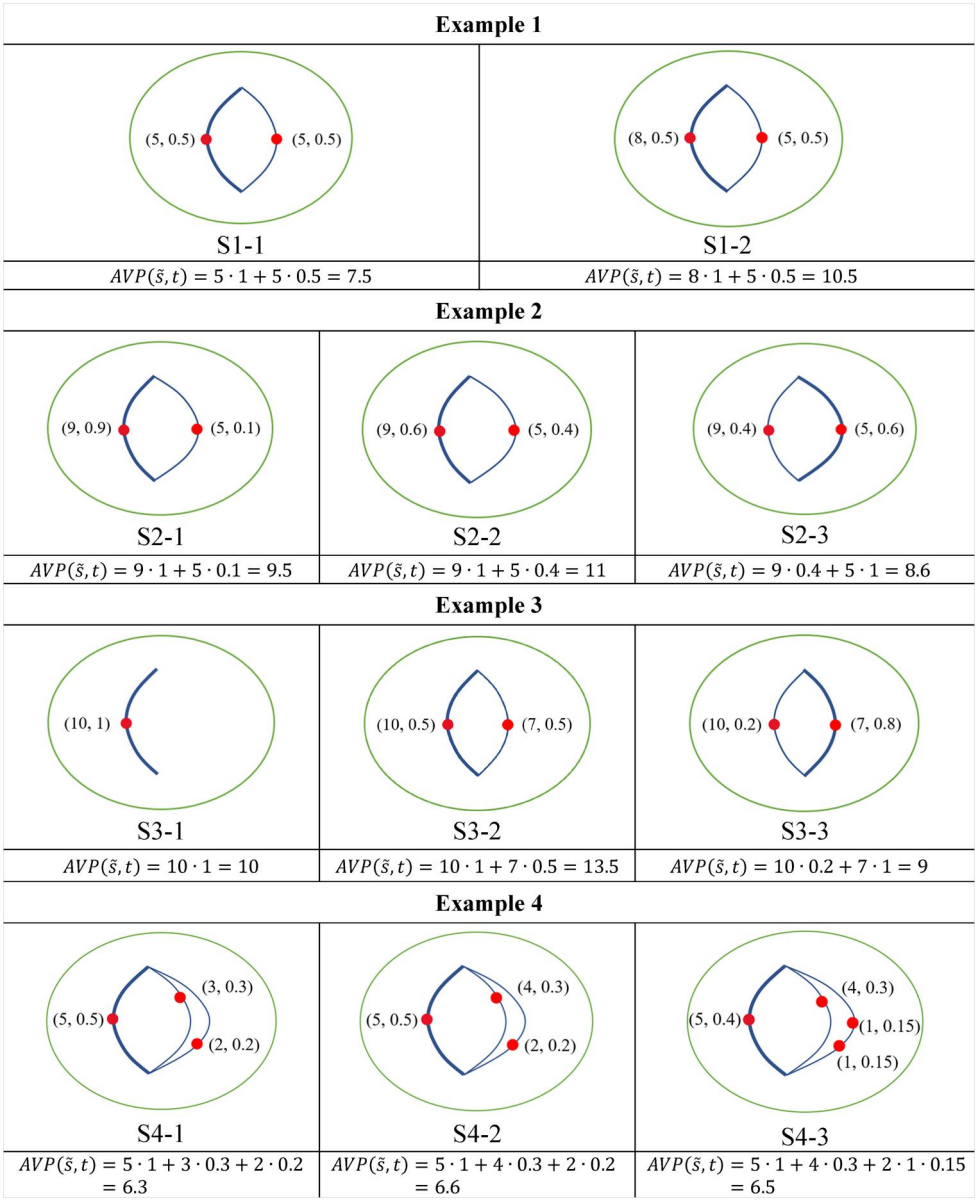
Examples of accessibility for the four remarks.

The suggested measure, Equation (20), reflects the realism that *AVP* secures the most likely visited locations as the core accessibility and supplements it with discounted contributions to accessibility from other locations in the heterogeneous interior of the STP. We use four small examples in Figure 2 to demonstrate the proposed accessibility measure. In all the subfigures, the green circle delineates the PPA and the red dots represent accessible activity locations situated on the blue paths linking the two anchor points. Each subfigure mimics an urban system. The pairs of attractiveness and visit probability, (G(e,t), P(e,t)), at activity state transition $\tilde{s}$ and time t are written next to the red dots. Specifically, the overweighed location in each system is indicated by the bold blue path. In each example, we compare the accessibility of two or three systems with variations in the number of activity locations, attractiveness, and visit probability. Below each subfigure, the accessibility is calculated according to Equation (20). The four remarks are illustrated by the four examples correspondingly.

In Example 1, S1-2 has higher accessibility since an activity location possesses higher attractiveness, keeping else unchanged. In Example 2, S2-2 has the highest accessibility, while S2-3 gets worse off due to a decrease in visit probability for reaching a more attractive location. S3-2 exhibits the greatest accessibility in Example 3, while the accessibility is worse off in S3-3 due to the addition of a location with a higher visit probability but less attractiveness. In Example 4, a trade-off needs to be made considering the location attractiveness, the interrelated visit probabilities, and accessible locations, which leads to a higher accessibility of S4-2.

Comparisons between the proposed accessibility measurement *AVP* (Equation (20)) and *NAL*, *AFT*, and *MFT* at $\tilde{s}$ and t (Equations (14)–(16)) in the four examples are shown in Table 1. The results demonstrate the advantages of *AVP*. In Example 1, *NAL* only emphasizes the effect of the number of accessible locations but ignores the location attractiveness, which cannot distinguish the accessibility in the two systems. In Examples 2 and 3, *AFT* and *MFT* neglect the heterogeneous STP interiors. In Example 4, *NAL*, *AFT,* and *MFT* make no trade-off among the accessible location number, attractiveness, and visit probabilities, resulting in biased or indistinguishable measurements of accessibility. *AVP* integrates and extends Equations (14)–(16) through the transformation of $P(e_{i},t)$ and simultaneously capture spatial heterogeneity by incorporating visit probabilities. However, it should be noted that other transformations in occasions may lead to different accessibility results. As a side note, as well-documented (Miller 2005, Neutens *et al*. 2010), the gravity-based accessibility measures are typically place-based and examine the physical proximity to various services from a specific location (e.g. home). Gravity-based measures can incorporate the attractiveness of the activity locations and the cost of physical separation from the reference location, expressed by a mode-specific distance-decay function. In contrast, the proposed *AVP* is a people-based measure that considers not only physical proximity but also focuses on individual-specific activity-travel patterns in space and time. Although gravity-based accessibility measures can be extended to incorporate simple spatial constraints, they have limitations in capturing individual heterogeneities due to their simplistic assumptions about spatial interaction and travel behavior (see page 6 in the Supplementary document for illustrating the comparison between *AVP* and gravity-based accessibility measures).

**Table 1.** Highest accessibility systems in the four examples.

Example No.	*NAL –* Equation (14)	*AFT* – Equation (15)	*MFT* – Equation (16)	*AVP –* Equation (20)
1	S1-1/S1-2	S1-2	S1-2	S1-2
2	S2-1/S2-2/ S2-3	S2-1/S2-2/S2-3	S2-1/S2-2/S2-3	S2-2
3	S3-2/S3-3	S3-2/S3-3	S3-1/S3-2/S3-3	S3-2
4	S4-3	S4-2/S4-3	S4-1/S4-2/S4-3	S4-2

## 3. Experimental results

**Figure 3.**
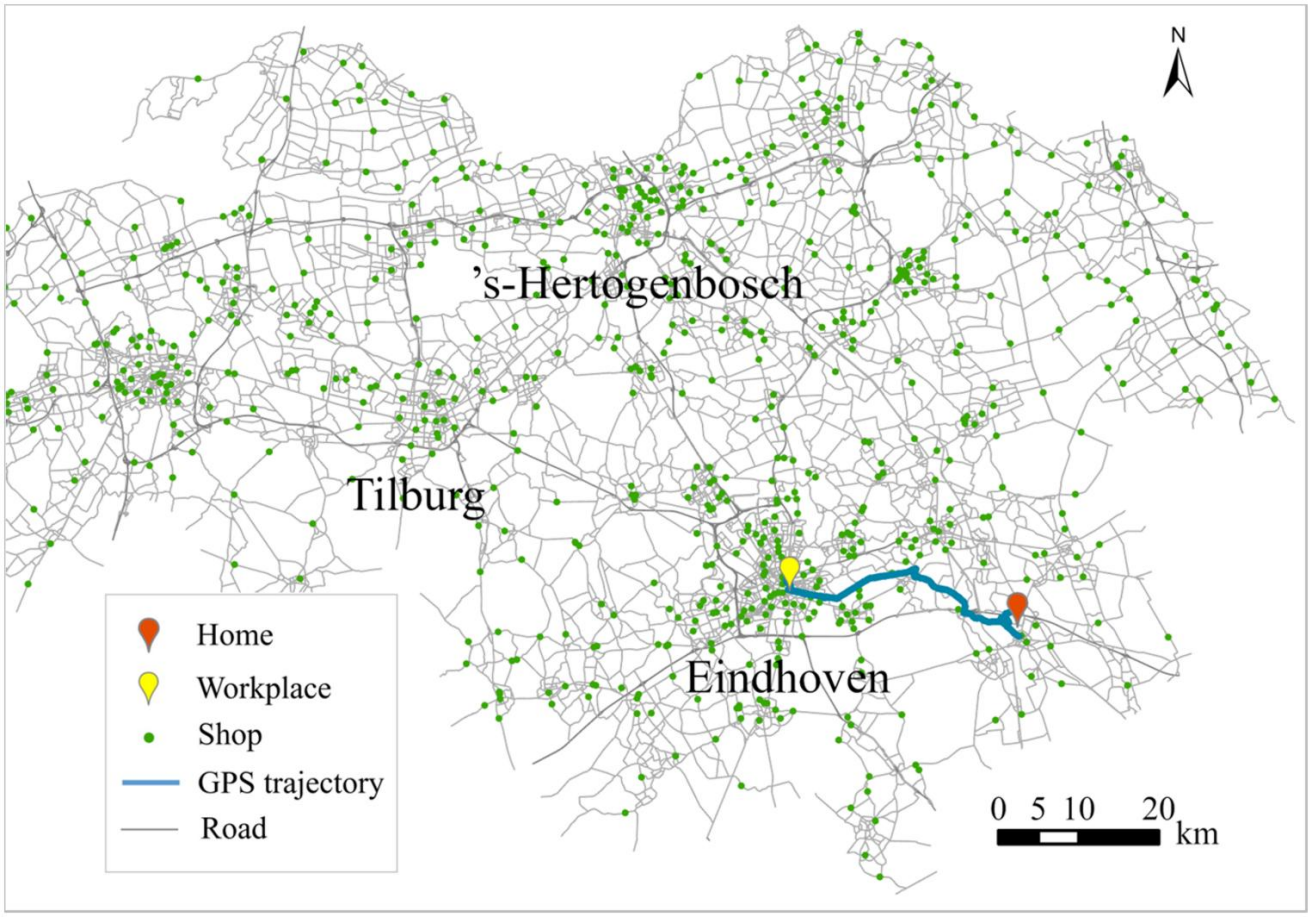
Study area and a selected individual AP.

To illustrate the proposed method for constructing the probabilistic STP and accessibility measure, we consider the North-Brabant Province, the Netherlands as the experimental study area (Figure 3). We use a sample of 2,714 individuals’ daily GPS trajectories with one work activity at a fixed workplace, one shopping activity with flexible locations, and a private car as the transportation mode to estimate the holding time density functions, which are processed from a GPS trajectory dataset collected by the ‘B-Riders program’ in 2014 in the Netherlands. Then, we arbitrarily select an individual to demonstrate visit probability and the proposed space–time accessibility measure. Detailed settings are as follows.

1. For each individual’s AP, the start time uses the recorded departure time from home. Based on empirical findings, the average proportion of standard deviation in the mean travel time during peak hours ranges from 0.2 to 0.35 (Eliasson 2006, Fosgerau 2016). Since the implementation of an AP involving multiple activities typically requires a longer period that is mostly allocated during non-peak hours, the time budget is set with a 10% extra by rule of thumb over the out-of-home time expense in the GPS trajectory. The start time and time budget together define the time window.
2. 809 Flexible activity locations are extracted as alternative locations for the shopping activity, consisting of one flexible activity location selected from the OpenStreetMap for each 4-digit postcode area and those shopping locations recorded in the GPS trajectories.
3. The road network has 47,901 nodes and 100,581 directed links. The roads are categorized into motorways, provincial roads, and local roads. The maximum attainable car speeds for peak hours ([7:00, 9:00] and [16:30, 19:00]) and non-peak hours on motorways, provincial roads, and local roads are set to <70, 50, 30> and <100, 80, 50> km/h, respectively.
4. The visit probabilities are calculated for each activity state every 5 min during peak hours and 30 min during non-peak hours. For travel links, four activity states are considered, including ‘neither work nor shopping is conducted’, ‘only work is conducted’, ‘only shopping is conducted’, and ‘both are conducted’, respectively; for activity links, two state transitions are considered, i.e. shopping before or after work.

### 3.1. Estimation results

For estimating the holding time density functions, individuals’ attributes of gender and age are selected as the socio-demographic variables in the latent class membership function based on data availability and privacy issues. Gender is dummy-coded considering male as the base level. Since the available GPS trajectories were collected from respondents with ages predominantly ranging from 45 to 70, individuals’ ages are categorized into three groups, which are less than or equal to 50, between 50 and 65, and greater than 65, respectively. Using dummy coding, the age group greater than 65 is the base level. According to the estimation methods discussed in Section 2.2, the parameters of the holding time density functions for travel links and activity links in *SNK* are estimated.

#### 3.1.1. Holding time function estimation for travel links

**Figure 4.**
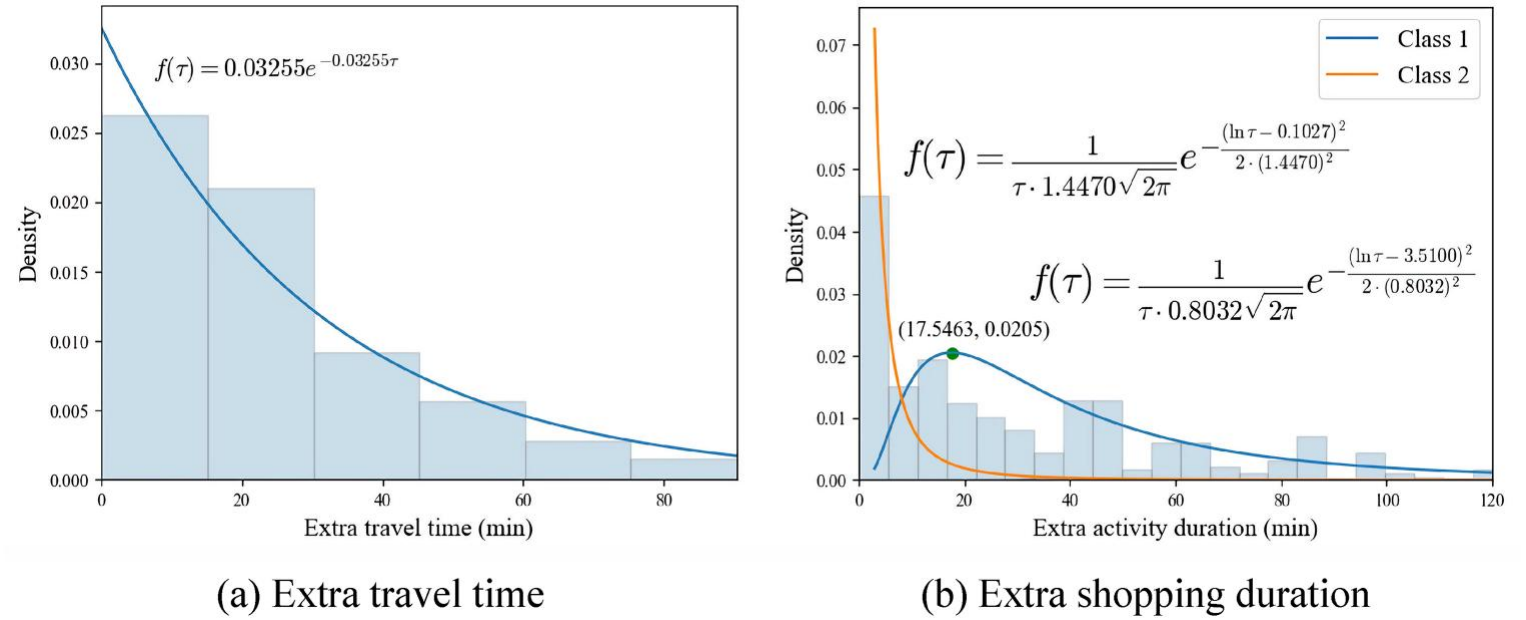
Histogram and the estimated distributions for travel and shopping.

According to Equations (5) and (6), $f_{e}^{q}(\tau )$ is estimated with 1 to 5 latent classes to determine the optimal number of latent classes. Table 2 reports the Log-likelihood, Bayesian Information Criteria (BIC), and Akaike information criterion (AIC) values with various classes. The optimal number of latent classes is determined based on the BIC, which imposes more penalties on complexity, as indicated by the number of estimates, compared to AIC. Corresponding to the lowest value of BIC, one class is identified for $f_{e}^{q}(\tau )$ for travel links. The values of log-likelihood show a marginal increase with the addition of more latent classes, which also indicates insignificant heterogeneity of multiple latent classes. The estimated parameter is λ=0.03255, which is found to be significantly different from zero at a 5% significant level (*p*-value < 0.001), indicating a good fit to the data as shown in Figure 4(a).

**Table 2.** Log-likelihood, AIC, and BIC with latent classes for travel.

Number of classes	Number of parameters P	Log-likelihood ln (L)	AIC	BIC
1	5	−6765.672654	13541.3453	13568.0072
2	10	−6765.672638	13551.3453	13604.6690
3	15	−6765.672623	13561.3453	13641.3308
4	20	−6765.672608	13571.3452	13677.9926
5	25	−6765.672593	13581.3452	13714.6544

AIC=2P−2ln (L); BIC=Pln (Q)−2ln (L). For the LR test, the null hypothesis: $\lambda_{k}=\frac{Q}{\sum_{q=1}^{Q}\tau_{q}},$ $\beta_{k}=0.$

#### 3.1.2. Holding time function estimation for shopping

For the flexible shopping activity, according to Equations (7) and (8), Table 3 shows the Log-likelihood, BIC, and AIC values with 1 to 5 numbers of latent classes. Corresponding to the lowest value of BIC, two latent classes are identified for the holding time density function. A likelihood ratio (LR) test was used to illustrate the goodness of fit of the estimated latent class model. Given two classes, the LR test has a significant p-value (<0.001), suggesting that the estimated two latent class lognormal distribution $f_{e}^{q}(\tau )$ provides a significantly better fit to the data than the null model for shopping as shown in Figure 4(b).

**Table 3.** Log-likelihood, AIC, and BIC with latent classes for shopping.

Number of classes	Number of parameters P	Log-likelihood ln (L)	AIC	BIC
1	6	−1522.065936	3056.1319	3079.0345
2	12	−1447.648105	2919.2962	2965.1015
3	18	−1433.337018	2902.6740	2971.3820
4	24	−1420.237746	2888.4755	2980.0862
5	30	−1412.163766	2884.3275	2998.8409

For the LR test, the null hypothesis: $\mu_{k}=\frac{1}{Q}\sum_{q=1}^{Q}\text{ln}\;\tau_{q},$ $\sigma_{k}^{2}=\frac{1}{Q}\sum_{q=1}^{Q}{\left(\text{ln}\;\tau_{q}-\frac{1}{Q}\sum_{q=1}^{Q}\text{ln}\;\tau_{q}\right)}^{2},$ $\beta_{k}=0.$

The estimation results are reported in Table 4. As illustrated by the estimated $\mu_{k},\sigma_{k},$ the extra shopping duration in latent class 1 tends to be longer with moderate variability compared to latent class 2. In terms of the membership function, the results of the estimated $\beta_{k}$ indicate that females are more likely to belong to class 1 preferring more extra time on shopping, given class 2 as the baseline latent class. The estimated parameters for age suggest that, compared to the group of age over 65, individuals aged between 50 and 65 are significantly more likely to belong to class 1, as indicated by a coefficient of 0.5751 with a *p*-value of 0.0113. Conversely, individuals aged under 50 do not show a statistically significant difference in the likelihood of belonging to class 1, as reflected by a coefficient of −0.1044 with a *p*-value of 0.7026. It should be noted that the choice of bin number or width in Figure 4 does not affect the estimation but is intended to better illustrate the underlying data for plotting the histogram (see page 7 in the Supplementary document for illustrating the histograms with different bin widths).

**Table 4.** Parameter estimates: shopping.

Class	Latent class size	$\beta_{k}$ (*p*-Value)		$\mu_{k},\sigma_{k}$ (*p*-Value)	
1	0.69	Constant	0.2631 (0.2015)	$\mu_{1}$	3.5100 (<0.001)
		Gender	0.4437 (0.0342)		
		Age ≤50	−0.1044 (0.7026)	$\sigma_{1}$	0.8032 (<0.001)
		50 < Age ≤65	0.5751 (0.0113)		
		Age >65 (base level)	0		
2	0.31	Constant	0	$\mu_{2}$	0.1027 (0.5762)
		Gender	0		
		Age ≤50	0	$\sigma_{2}$	1.4470 (<0.001)
		50 < Age ≤65	0		
		Age >65 (base level)	0		

### 3.2. Results of visit probability

The simulated probabilistic STP delimits the accessible travel links and activity locations for a daily AP. The AP of an arbitrary individual who lives in the Eindhoven area is extracted from the GPS trajectories. Given the recorded departure time $t_{H_{0}}=7:20,$ the time budget as 742 min, and also the individual’s minimum duration of working and shopping as 558 and 36 min respectively, the *SNK* is constructed based on the road network and the selected activity locations, for constructing the activity-based STP with out-of-home time window [7:20, 19:42].

#### 3.2.1. Travel links

**Figure 5.**
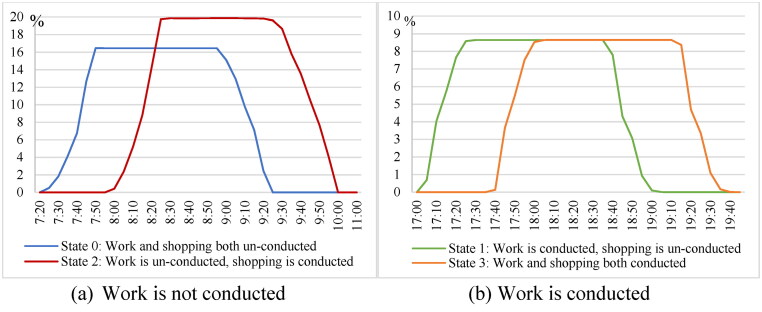
Percentage of accessible travel links within the STP.

**Figure 6.**
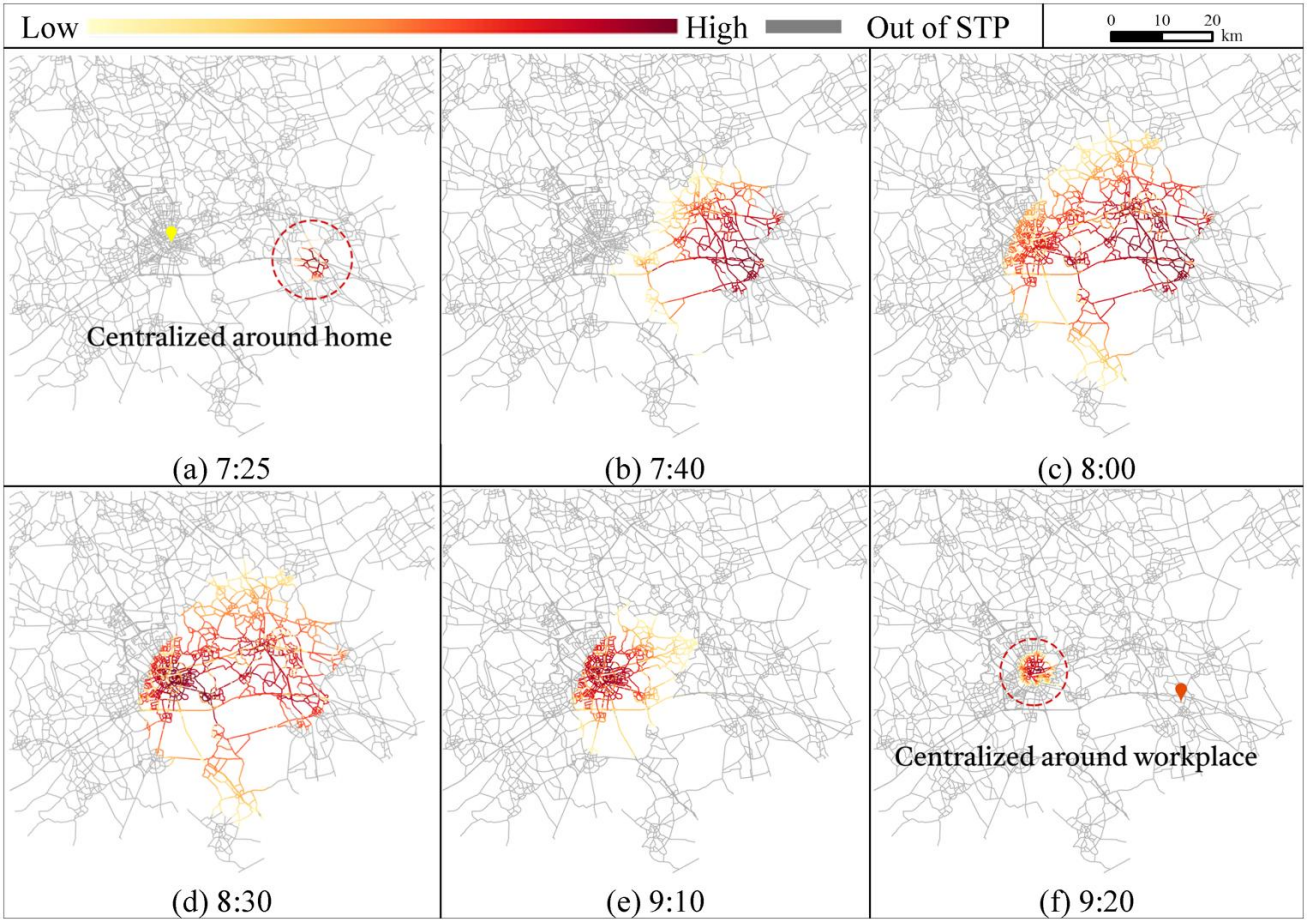
Planar STP interior of travel links at activity state 0 (none is conducted).

**Figure 7.**
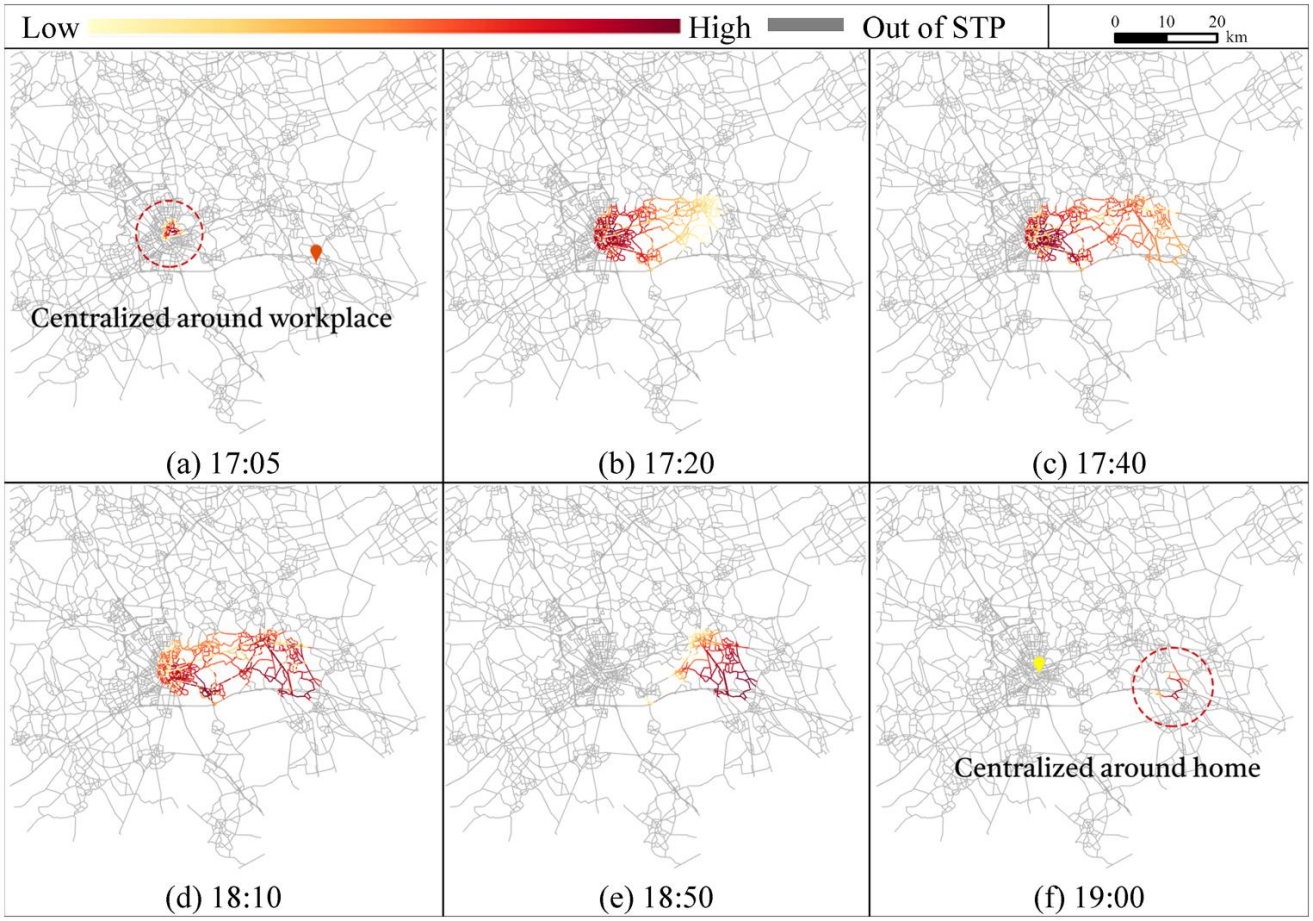
Planar STP interior of travel links at activity state 1 (only work is conducted).

Considering flexible activity chains, we show the visit probabilities for conducting the flexible activity. The results in Figure 5 show the percentages of accessible travel links within the STP at different activity states and time points. Figures 6 and 7 further illustrate the probabilistic interior of the STP for travel links at activity states 0 and 1 for conducting shopping before and after work, respectively. Representative time points are selected to better demonstrate the probabilistic STP interior over time.

At activity state 0, none of the activities is conducted, and the individual departs from home at 7:20. As shown in Figure 5(a), the number of travel links within the STP (blue curve) increases given sufficient available time during [7:20, 7:55] and decreases during [8:55, 9:25] since work has to be done with a fixed duration (558 min from the trajectory). The accessibility percentage peaks during [7:55, 8:55], reflecting the maximum 16.46% of travel links that can be accessed within the STP given the time budget. At state 2 where only shopping is conducted (red curve in Figure 5(a)), the STP starts to include travel links around home at 8:00 after shopping with a duration of 36 min is conducted within [7:20, 8:00]. After 9:15, the accessible travel links decrease to start working at the workplace. The number of accessible travel links is larger at state 2 than that at state 0 because more travel links are explored around home for shopping before work within the available time. There are no accessible travel links during [10:00, 17:00] since the individual is working. At state 1 where only work is conducted (Figure 5(b)), the individual travels to conduct shopping after 17:00. Compared to the results at state 3 both the working and shopping are completed (orange curve), the increasing and decreasing of the accessible travel links occurs about 36 min early (green curve), to ensure shopping can be conducted before arriving at home at 19:42.

To explore the interior of the probabilistic STP, Figure 6 shows the visit probability distributions of the travel links at activity state 0 for conducting the activities. The time points for illustration are selected with intervals of 15, 20, 30, and 40 min from 7:25 (note that no travel link is accessible at 7:20). The scale of the visit probabilities with a color scheme in each Figure depends on the current activity state and time moment. To conduct shopping before work, the travel links centered around home first have high visit probabilities at 7:25 (Figure 6(a)). During [7:25, 9:20], the links with high visit probabilities (darker red) gradually transfer from the home-centered area to the workplace-centered area (Figure 6(b–e). At 9:20, only travel links centralized around the workplace can be accessed (Figure 6(f)). The travel links far from the central area around the fixed activity locations are less likely to be visited due to the time constraint.

At activity state 1, time points for illustration are selected with intervals of 15, 20, 30, and 40 min within [17:05, 19:00]. For conducting shopping, the travel links with high visit probabilities are centralized around the workplace immediately after work is conducted at 17:00 (Figure 7(a)) and then expand from the workplace towards home (Figure 7(b–e)). At 19:00, only the travel links near home are accessible (Figure 7(f)), since the individual has to complete shopping and return home no later than 19:42.

As the results show, the visit probability distributions of travel links within STP vary over different activity states and time moments. The STP interior over states and time collectively reflects the individuals’ potential behavior of conducting the flexible activity subject to space–time constraints.

#### 3.2.2. Flexible activity locations

**Figure 8.**
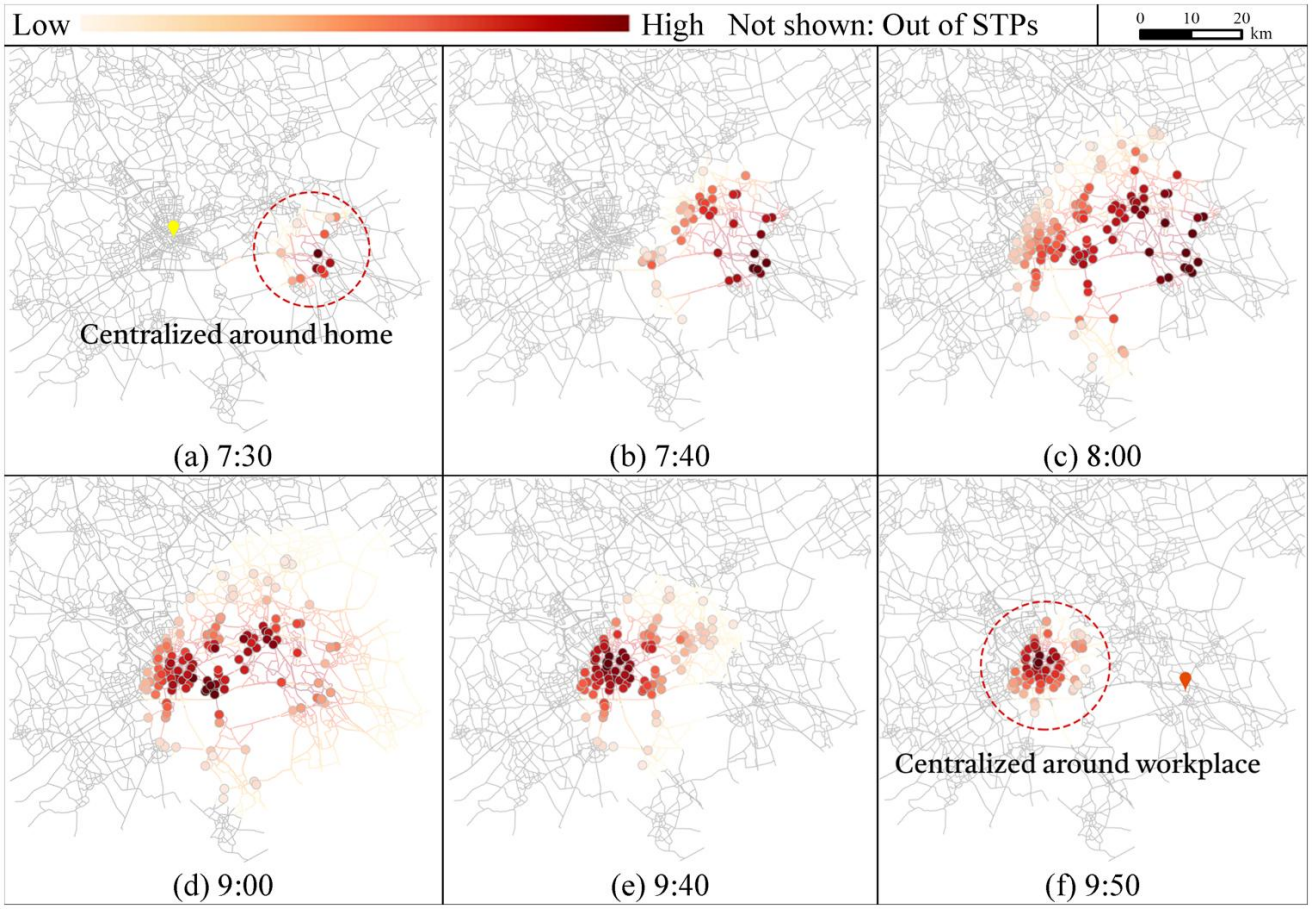
Planar STP interior of flexible activity locations before work ($\tilde{s}:$ 0→2).

**Figure 9.**
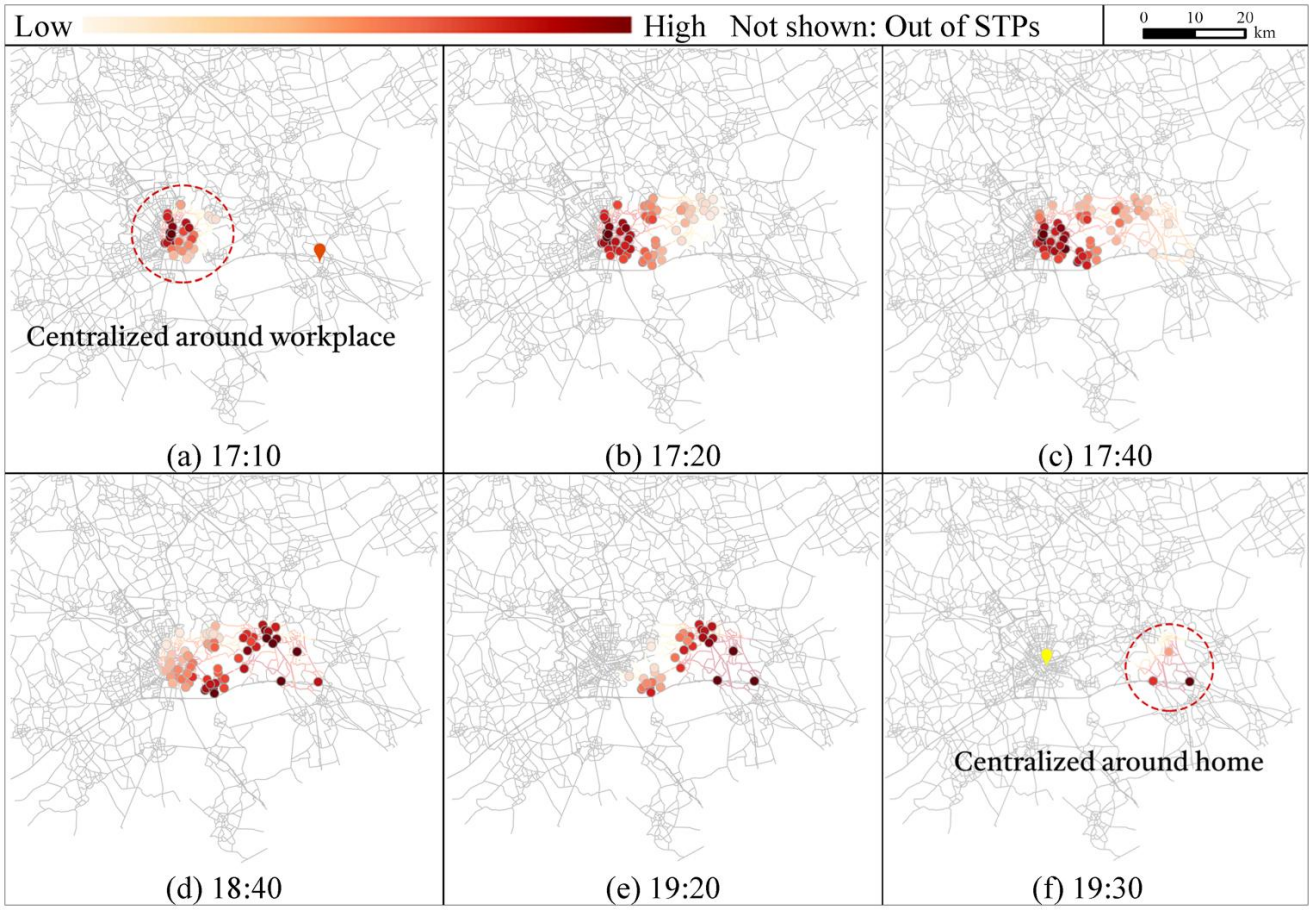
Planar STP interior of flexible activity locations after work ($\tilde{s}:$ 1→3).

Figures 8 and 9 illustrate the visit probabilities of activity locations for shopping over different time points and activity state transition due to flexible activity sequences. For shopping before work ($\tilde{s}:$ 0→2), the number of accessible locations increases after 7:20. The representative time points are selected within [7:25, 9:55] after 7:20. At 7:30, only 13 locations can be accessed centralized around home (Figure 8(a)). During [7:25, 7:50], more locations are accessible but those near home still have relatively higher visit probabilities (Figure 8(a,b)). Within [7:55, 9:30], the number of accessible activity locations remains unchanged, but the shopping locations with high visit probabilities gradually shift from the area around home to the workplace (Figure 8(c,d)), and finally centralized around the workplace during [9:30, 9:55] (Figure 8(e,f)).

For shopping after work ($\tilde{s}:$ 1→3), as shown in Figure 9 with representative time points within [17:05, 19:42], the accessible shopping locations with high visit probabilities expand from the center of the workplace during [17:05, 17:30] (Figure 9(a,b)), then gradually shift (Figure 9(c,d)), and finally concentrate around home before 19:42 (Figure 9(e,f)).

The results reflect that the flexible activity locations that provide more spatial and temporal flexibility for the AP are more likely to have higher probabilities of being visited, and the visit probabilities differ significantly over activity sequences and time.

### 3.3. Results of accessibility measurements

The space–time accessibility within the probabilistic STP is simulated using Equations (19) and (20). We define the location attractiveness $G(e,t)=t_{{j|}_{s^{\prime }}}^{+}-t_{{i|}_{s}}^{-}-d_{\alpha }^{\text{min}}$ as the flexible time for each $e\in \zeta (\tilde{s},t).$ The calculated P(e,t) is normalized with $\sum_{e}P(e,t)=1$ at different $\tilde{s}$ and t. To examine the validity of the proposed measure, we compare $\text{AVP}(\tilde{s},t)$ with the indicators of *NAL*, *AFT* (h), and *MFT* (min) for each $(\tilde{s},t),$ which are calculated by Equations (14)–(16), respectively at each $\tilde{s}$ at t.

**Figure 10.**
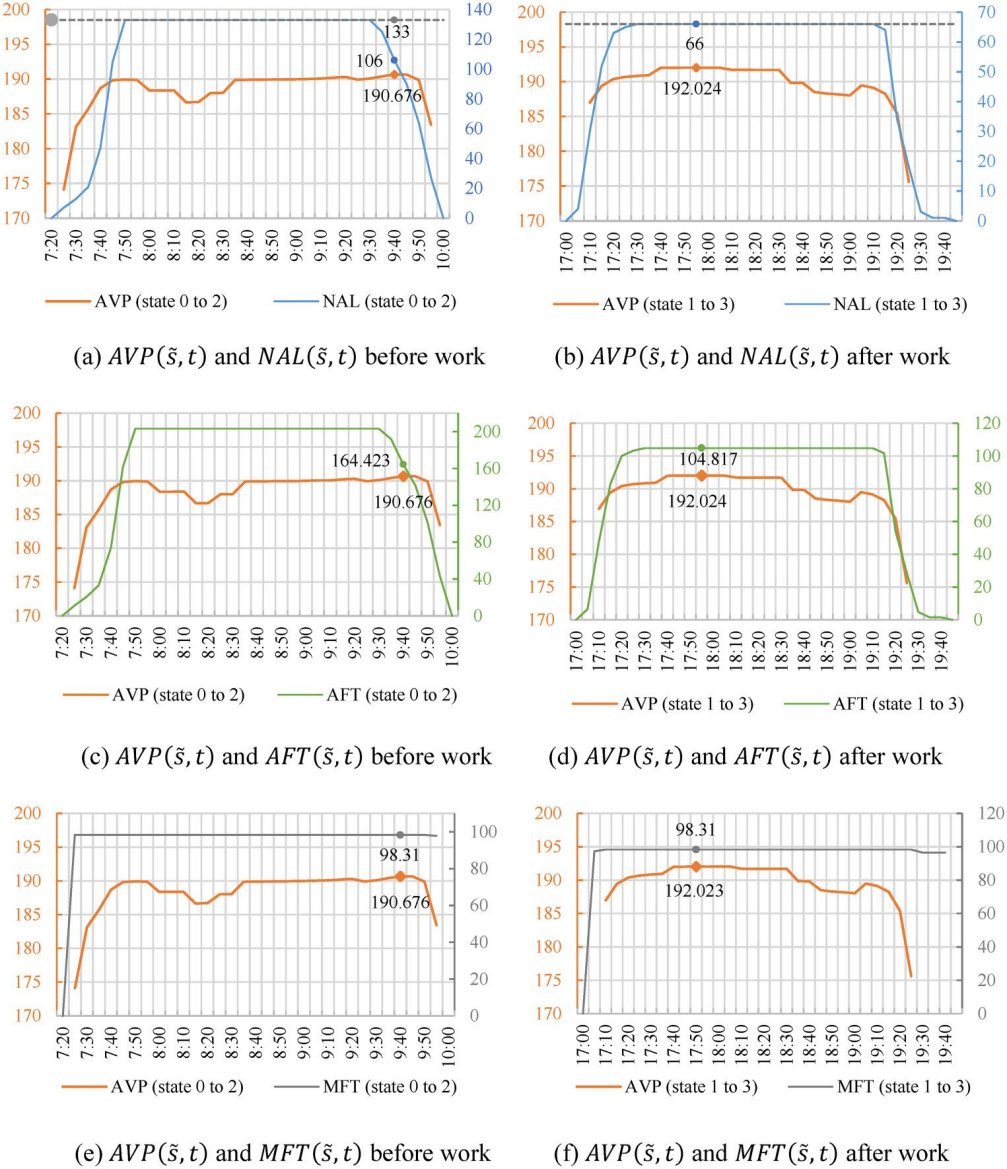
Comparisons of $\text{AVP}(\tilde{s},t),$ $\text{NAL}(\tilde{s},t),$ $\text{AFT}(\tilde{s},t),$ and $\text{MFT}(\tilde{s},t).$

**Figure 11.**
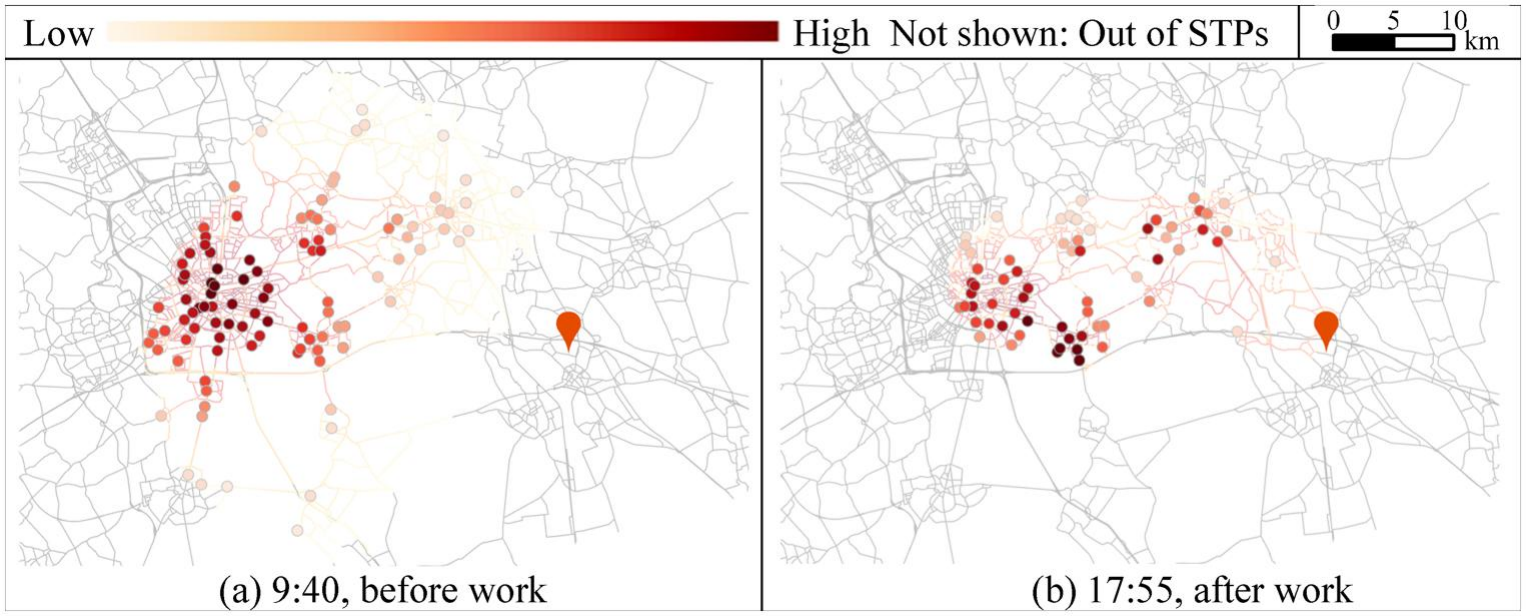
Flexible activity locations within STPs with maximum accessibilities.

Figure 10 illustrates the comparison between *AVP* and *NAL*, *AFT*, and *MFT*, respectively. Subfigures in each row show the accessibility to shopping locations before and after work at each $\tilde{s}$ and t for each pair of comparisons. Figure 11 shows the STP interior at the time moments of the maximum accessibility before and after work, to explore the effect of visit probability on accessibility. For shopping before work ($\tilde{s}:$ 0→2), as shown in Figures 10(a,c,e) and 11(a), the maximum $\text{AVP}(\tilde{s},t)$ appears at 9:40 when the activity locations with high visit probabilities centralize around the workplace, illustrating that locations with more flexible duration and visit probabilities contribute to higher accessibility. In comparison, if using *NAL* as the measure, only 106 locations can be accessed within the STP at 9:40, which is less than the maximum *NAL* of 133 (Figure 10(a)). The values of *AFT* have the same tendency indicating a lower accessibility at the moment (Figure 10(c)). *MFT* only considers the maximum flexible time, resulting in a lack of variations in accessibility over time (Figure 10(e)). The local minimum $\text{AVP}(\tilde{s},t)$ appears at 8:15 when the values of *NAL*, *AFT*, and *MFT* reach the maximum with 133 activity locations accessible. *NAL*, *AFT*, and *MFT* evaluate the accessibility to be higher at the moment because the increased number of accessible locations leads to more dispersed visit probabilities.

After work is conducted ($\tilde{s}:$ 1→3), in Figures 10(b,d,f) and 11(b), the maximum $\text{AVP}(\tilde{s},t)$ appears at 17:55 when most of the 66 accessible locations near the workplace have relatively higher visit probabilities than the ones near the home, indicating that shopping after work at the locations near the workplace is more likely and contributes to higher accessibility. The result of *AVP* is consistent with *NAL*, *AFT*, and *MFT* since the accessible locations with higher visit probabilities at this moment also possess more flexible time. Compared to the measurement of *NAL* in Liao (2021), which are 133 and 66 for conducting shopping before and after work (gray dotted line in Figure 10(a,b)), the proposed measurement provides more information reflecting the STP interior.

All in all, without considering the probabilistic interior of STP, *NAL*, *AFT*, and *MFT* may introduce biases in evaluating the accessibility to reach opportunities.

## 4. Conclusions and future work

This study proposes a model framework to construct the probabilistic STP of an AP with flexible activity chains based on the multi-state supernetwork representation. For capturing the individual heterogeneity, latent class models are formulated and estimated for travel and activity participation using daily mobility trajectories. Incorporating visit probabilities, a space**–**time accessibility measurement *AVP* is proposed, which is validated as a comprehensive accessibility measure. The results of the example illustrate that the visit probabilities of travel links and activity locations over different activity states and time points can describe the interior of the STP. Meanwhile, the latent class models of extra time for traveling and conducting activities capture the individuals’ heterogeneity. The comparisons between the *AVP* and three traditional accessibility measures demonstrate the advantage of *AVP* in measuring accessibility considering heterogeneous STP interiors.

The construction of the STP for daily APs is a research field that only has few explorations, presenting opportunities for further investigation. Based on the current study, we discuss the following limitations, which should be addressed in future work. First, from the data and estimation aspect, the analysis of the latent class models is limited by two socio-demographic variables along with the available GPS data. An enriched GPS trajectory dataset should provide more choices of the socio-demographic characteristics for defining the population groups. Second, from the experimental simulation aspect, as experimental analyses in Section 3 are mainly for demonstration of the modeling feasibility, some settings and the assessment of accessibility require further empirical validations. Third, from the modeling aspect, further research should explore the probabilistic interior of the STP constructed over multi-modal and dynamic *SNK* since multimodality and transportation network dynamics are common phenomena.

Additionally, an extension of the proposed state-time dependent space**–**time accessibility measurement is the evaluation of transportation infrastructure to better connect individuals with opportunities for conducting APs characterizing human basic needs. Since the obtained visit probabilities can reflect individual heterogeneity, the models can be further extended to reveal disparities between different population groups. The constructed probabilistic STP of an AP can also be extended in the activity-based travel demand modeling, for example, by applying the estimated visit probabilities of travel links and activity locations in a dynamic choice set generation of ATPs to eliminate the choice set of routes and activity locations based on a probability threshold held by an individual.

## Supplementary Material

Supplemental Material

## Data Availability

The data and codes that support the findings of this study are available on figshare.com with the identifier(s) at https://doi.org/10.6084/m9.figshare.25257154.v1, where the Supplementary document can be found.
