# Supplementary material for: Visit probability and accessibility within space–time prism of activity program
Source: Int J Geogr Inf Sci. 2024 Jul 17;39(5):1076–99. doi: 10.1080/13658816.2024.2378066 (PMC11996065; doi:10.1080/13658816.2024.2378066)
Supplement: Supplemental Material [file TGIS_A_2378066_SM5406.zip › [TGIS_[2378066]_Supplementary Material] 01.pdf]

Supplementary document for manuscript:

“Visit probability and accessibility within space–time prism of activity program”

This document includes figures and explanations used for further understanding of the *SNK* representation, space–time prism (STP), and the accessibility measures.

The following abbreviations are used in this document:

SNK: Multi-state supernetwork
ATP: Activity-travel pattern
AP: Activity program
STP: Space–time prism
PPA: Potential path area
SMP: Semi-Markov process
AVP: Aggregate attractiveness with visit probability

Illustration of the trip-based and the activity-based STPs:

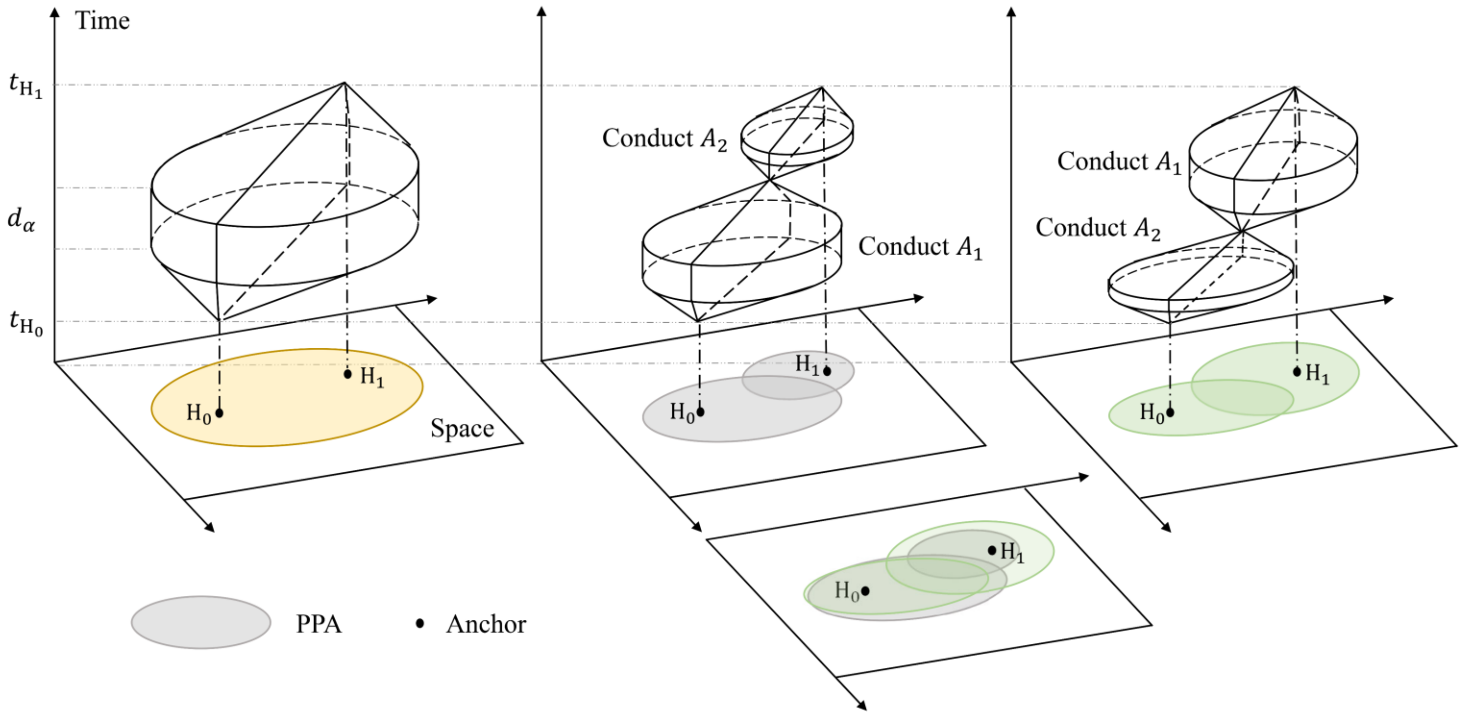

(a) A trip-based STP

(b) An activity-based STP

Figure S1. The comparison between the trip-based STP and activity-based STP.

Compared to a simple trip-based STP of conducting a single flexible activity (**Figure** S1 (a)), the activity-based STP (**Figure** S1 (b)) delimits the space–time opportunities associated with all feasible ATPs. (PPA: projection of united STPs)

The comparison of figures (a) and (b) demonstrates that the incorporation of activity-travel chains with flexible activity sequences introduces complexity and causes irregular geometrical structures into the construction of the STP and PPA.

Illustration of the *SNK* representation:

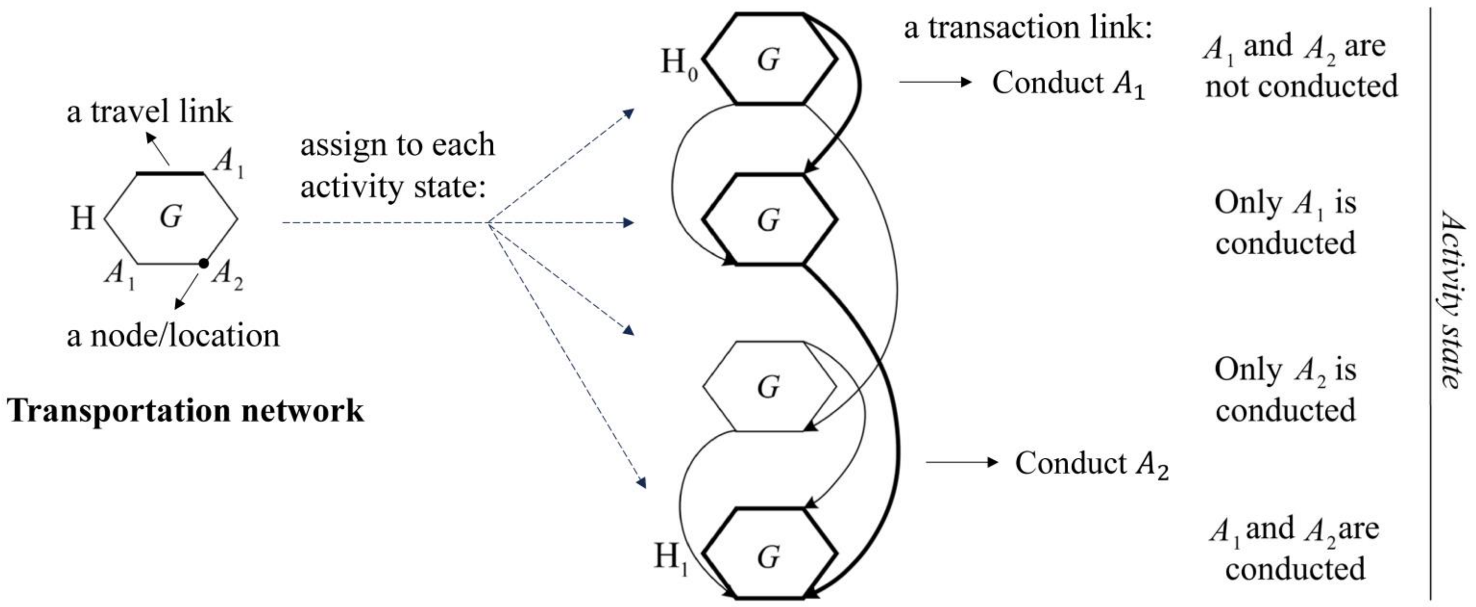

Figure S2. Multi-state supernetwork representation with a single mode.

H_0, H_1: origin and destination of an AP at first and last activity state.
A_1, A_2: two activities.

- activity state:
  explains which activities have been conducted.
- Transportation network *G*:
  models path choice to travel from O to D;
  assigned to each activity state.
- Transaction/activity link:
  interconnects the same activity location at different activity states. (bold link in **Figure** S2)
- AP implementation:
  decomposed into path choice through a network (*SNK*) of networks (transportation network *G*) of differentiated activity states. (bold path in **Figure** S2)

Illustration of the *SNK* representation:

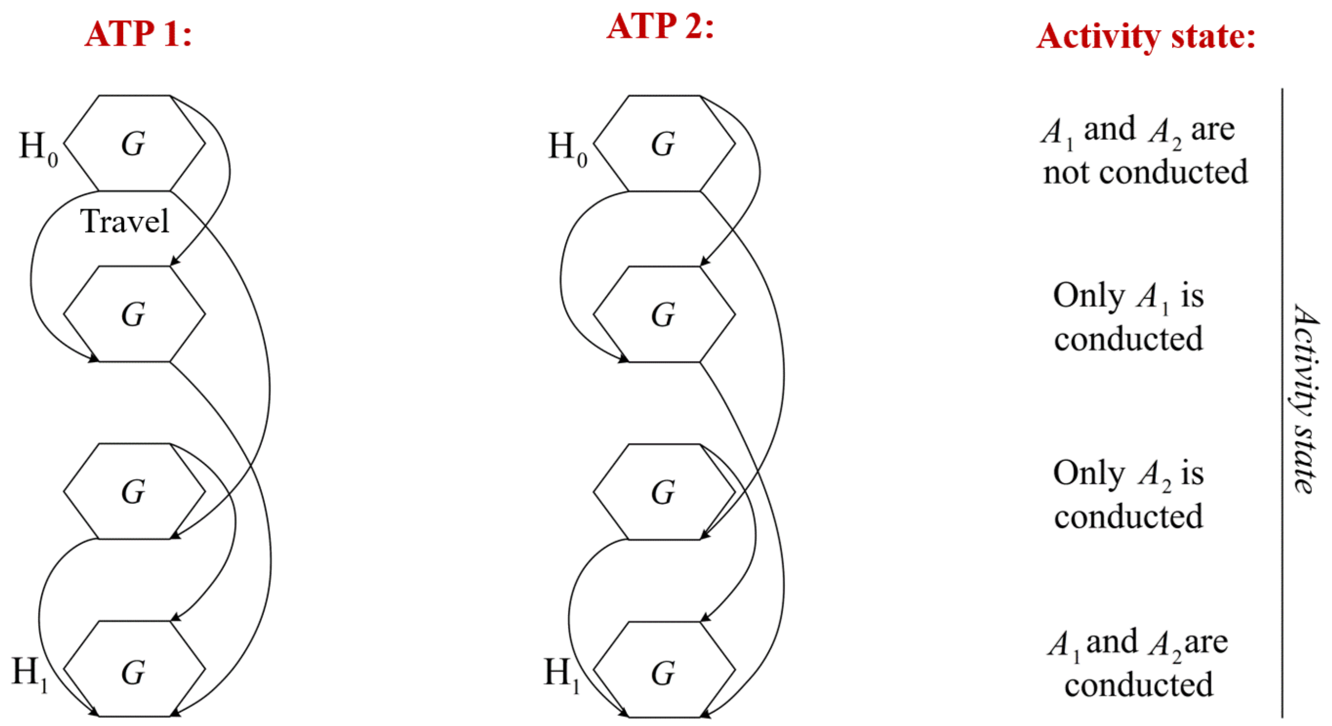

Animated **Figure** S3. Supplementary explanation of **Figure** S2: the implementation of an AP.

Given an assumption that activity A_1 has two locations and A_2 only has one, there are two activity links for conducting A_1 at two locations and a single activity link for A_2 for one episode of activity state changes, respectively.

Figure S3 illustrates two possible ATPs with different activity sequence:

- Left sub-figure:
  the generation of ATP 1 that conduct A_2 before A_1.
- Right sub-figure:
  the generation of ATP 2 that conduct A_1 before A_2.

Illustration of the visit probability model in *SNK*:

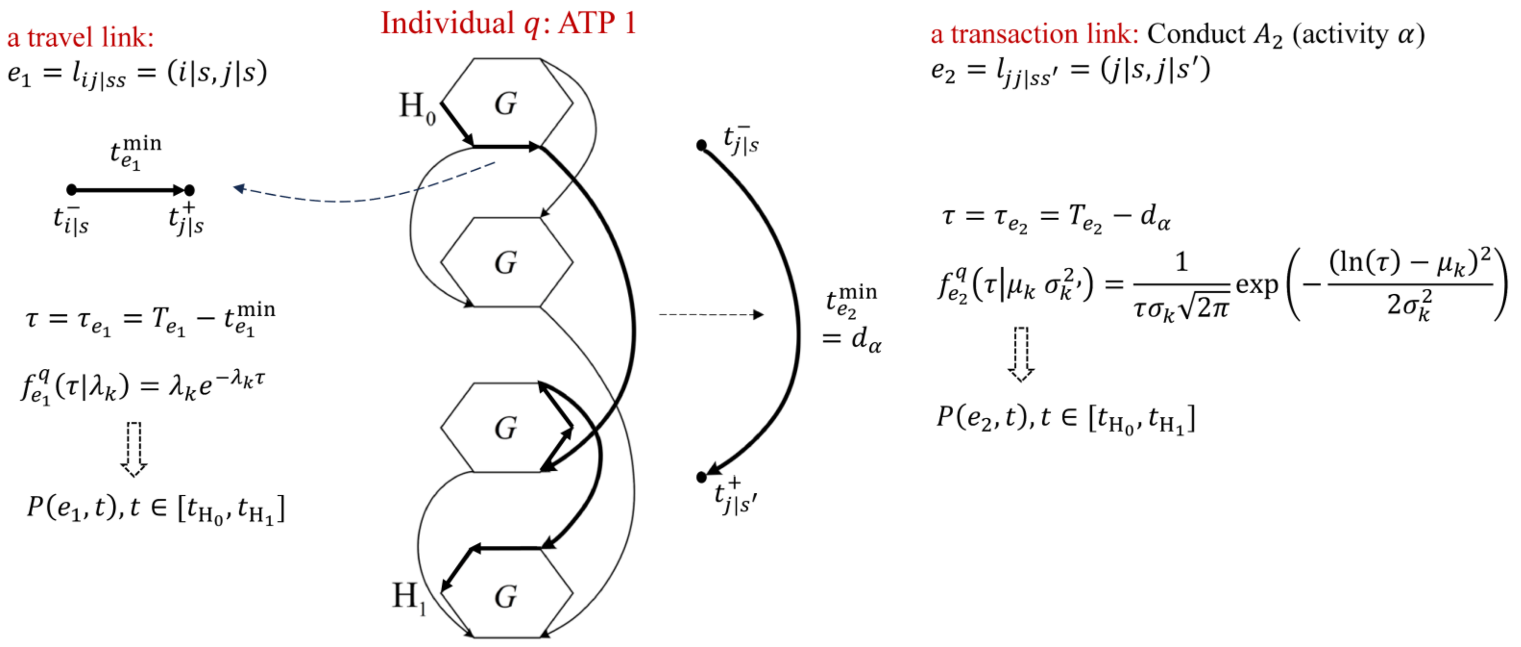

A generalized link: $e = l_{ij|ss'} = (i|s, j|s')$

SMP state: "a movement starting from node $i|_s$ to node $j|_{s'}$ along link $l_{ij|ss'}$ but has not arrived at $j|_{s'}$ yet.

Since both travel and activity participation are represented as links in *SNK*, the relevant definitions for the:

- Visit probability model (include SMP)
- Latent class models

are illustrated for travel links and activity links, respectively, in **Figure** S4.

Figure S4. Supplementary explanation of **Figure** S2: the definitions of visit probability model in *SNK*.

Illustration of the comparison between *AVP* and gravity-based accessibility measures:

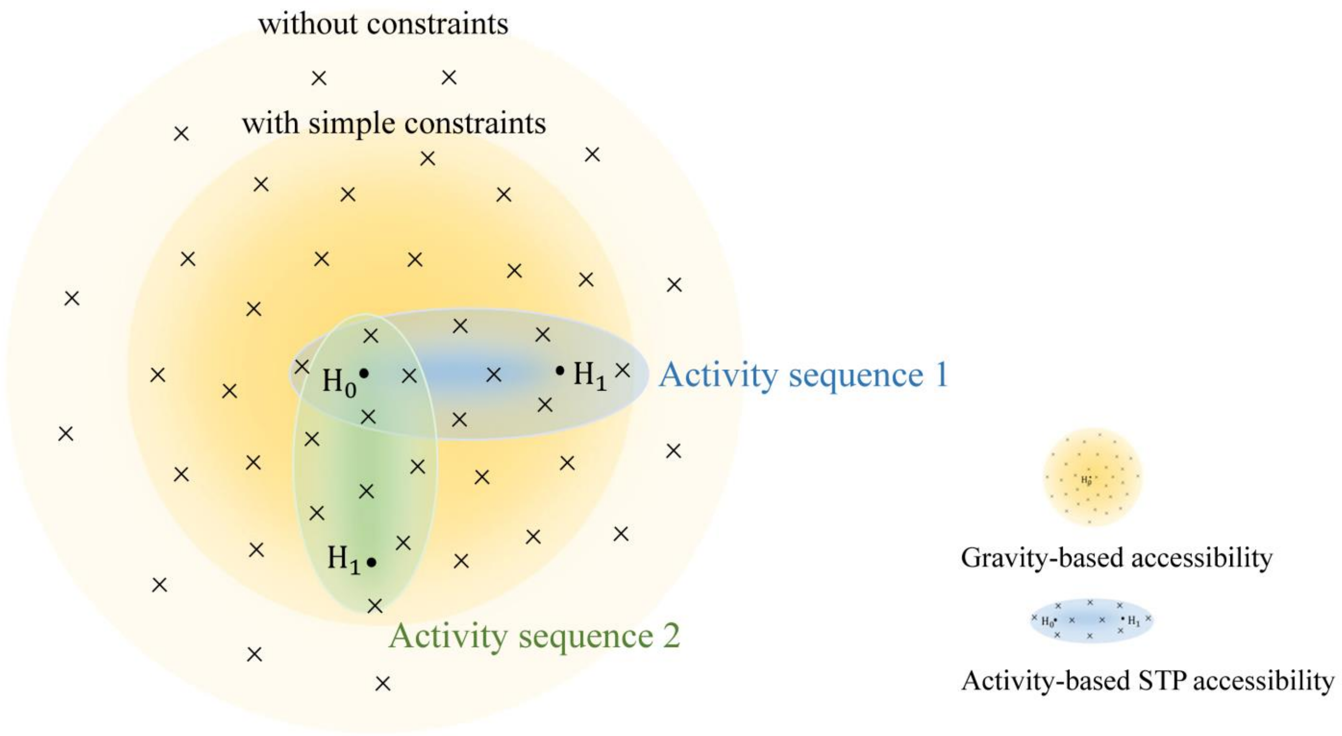

Figure S5. Differences between *AVP* and gravity-based accessibility measures.

H_0, H_1: two anchors (home) of an AP at different activity states
×: an accessible activity location within the constraints

Gravity-based accessibility measures are typically **place-based** and examine the physical proximity to various services from a specific location (e.g., home). They have limitations in heterogeneities due to their simplistic assumptions about spatial interaction and travel behavior, compared to the **people-based** measures (e.g., *AVP* in this study) that also focus on individual activity-travel patterns in space and time.

As demonstrated in the figure:
gravity-based accessibility measures cannot fully examine the accessibility to the potential locations for completing complex activity-travel chains (with different activity sequences) under space-time constraints.

Illustration of the estimation results:

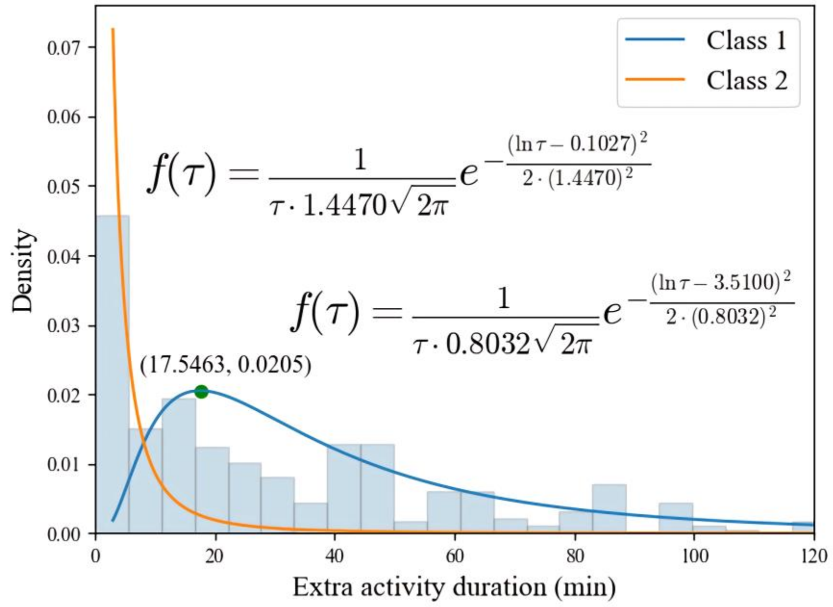

Used: Figure 4(b) histogram with interval width = 5 min

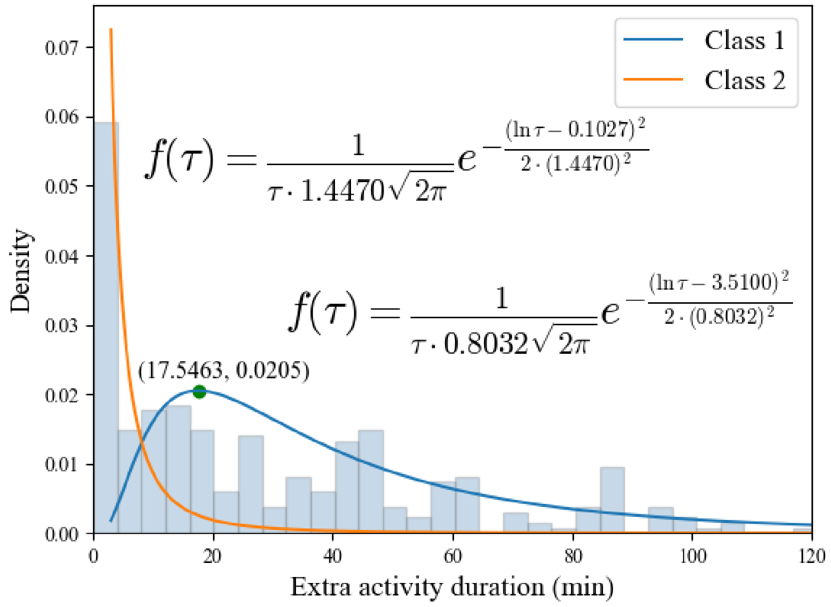

Tested: (i) histogram with interval width = 3.6 min

Figure S6. Histograms with different interval widths for shopping.

For plotting histogram of the original data, the choice of bin number or width does not affect the estimation but is intended to better illustrate the underlying data (as seen in **Figure** 4(b) used in the manuscript compared to the right-hand side figure).
