# Supplementary material for: Visit probability and accessibility within space–time prism of activity program
Source: Int J Geogr Inf Sci. 2024 Jul 17;39(5):1076–99. doi: 10.1080/13658816.2024.2378066 (PMC11996065; doi:10.1080/13658816.2024.2378066)
Supplement: Supplemental Material [file TGIS_A_2378066_SM5406.zip › [TGIS_[2378066]_Supplementary Material] 02.pdf]

Replication package explanatory file

Manuscript title: Visit probability and accessibility within space–time prism of activity program

Keywords: CPP, Python, OpenStreetMap, MLE estimation (EM algorithm)

Journal Name: International Journal of Geographical Information Science

Data description: All datasets are stored in the corresponding code file folders, the structures of the projects are illustrated below.

C++ Code for Visit Probability Simulation

Code description:
The program was written in C++ and tested in Visual Studio 2019. It should be possible to run the program in different environments as long as the necessary compiler and debugger are set. Only those important files for producing final results are explained. Necessary explanations for functions are written within the scripts.

The C++ codes are designed into 3 modules:
(1) Visit probability within space–time prism (STP) of an activity program (AP) simulation;
(2) Proposed accessibility measurement (*AVP*) calculation;
(3) Traditional accessibility measurements calculation.

Module (1) generates the STP for an AP of multiple activities with flexible sequences based on multi-state supernetwork (single mode: car), and simulates the visit probabilities of the travel links and activity links at different time points and activity states (transitions).
Module (2) calculates the proposed accessibility measurement (*AVP*) for activity links of flexible activity locations within the STP.
Module (3) is a supplement to Module (2), calculates three traditional accessibility measurements (*NAL, AFT, MFT*) with *AVP*.

Considering that one may only want to run a single module, the three modules are coded with separate entry points and are designed to be executed sequentially, with Module (1) being essential to run first.

It should be noted that, Module (2) functions by reading the output files from Module (1) written to a directory, Module (3) performs calculations based on the output files from Module (2). One can choose whether to run model (2) and module

(3) or not by setting the:

`bool CalculateAccessibilityMeasuresNeeded`, or
`bool CalculateAMAFTNALMFTNeeded`

to "`true`" or "`false`" in `MainFunctions.cpp` (in order).

CPP Project Structure

```
/cppCodes
   /VisitProbability_STP_AP
      /VisitProbability_STP_AP (Project Folder)
         /VisitProbability_STP_AP.vcxproj
         /code.h
         /code.cpp
         /…
      /VisitProbability_STP_AP.sln
   /DataFiles
      /Sub Folder1
         /data1.txt
         /data2.csv
         /…
      / Sub Folder2
         /data3.csv
```

Table 1 Visit Probability Simulation datasets.

	filename	contents
1	NLNB_Nodes.txt	Nodes with X/Y coordinates of the transport network.
2	NLNB_Links.txt	Edges with information of length, road classification, bi-direction, travel speed, etc. of the transportation network.
3	NLNB_outATPs.csv	Individuals' daily mobility GPS trajectories for conducting APs.
4	NLNB_outATPs_startTimes.csv	Start time of the mobility GPS trajectories.
5	NLNB_FlexActLocs.csv	Flexible activity locations (old road network node ID) with activity type.
6	NLNB_FixedFlexActLocsNewNodeID_ATPID0_toPC.csv	Flexible activity locations (new road network node ID) with activity type and belonging postcode area.
7	NLNB_ATPsUserXiVec_3ageGroup_dummyCoding.csv	Individual's socio-demographic characteristics of the selected AP (dummy coding, 3 age group classification).
8	NLNB_VP_TimePoints.csv	Selected time points for simulation.
9	NLNB_TvlEXPDistParas_3ageGroup_c1.csv	Estimated distribution parameters of the exponential latent class model for travel links.
10	NLNB_TvlEXPBetaParas_3ageGroup_c1.csv	Estimated membership function parameters of the exponential latent class model for travel links.
11	NLNB_FlexActLogNDistParas_3ageGroup_c2.csv	Estimated distribution parameters of the log-normal latent class model for activity links (flexible).
12	NLNB_FlexActLogNBetaParas_3ageGroup_c2.csv	Estimated membership function parameters of the log-normal latent class model for activity links (flexible).

13	NLNB_FixedActLogNDistParas_3ageGroup_c3.csv	Estimated distribution parameters of the log-normal latent class model for activity links (fixed).
14	NLNB_FixedActLogNBetaParas_3ageGroup_c3.csv	Estimated membership function parameters of the log-normal latent class model for activity links (fixed).

Notes:

1. `NLNB_Nodes.txt` and `NLNB_Links.txt` are processed from the shapefiles of the road network of North-Brabant, the Netherlands, which can be downloaded from OpenStreetMap.
2. The original `NLNB_FixedFlexActLocsNewNodeID_ATPID0_toPC.csv` data can also be generated for a newly selected AP, by activating the codes of line 44-45 in `ConstructPVNSNK.cpp` with an added column of the spatial joined 4-digit postcode.

Table 2 Visit Probability Simulation C++ code files explanations.

	filename	descriptions
Main Function		
1	MainFunctions.h/.cpp	Main functions (entry) for:<br>1) Visit probability within STP of AP simulation<br>2) Proposed accessibility measurement calculation<br>3) Traditional accessibility measurements calculation
Multi-state supernetwork construction		
2	GPSElements.h/.cpp	Custom classes designed for GPS data management.
3	ATPInOut.h/.cpp	Custom classes designed for AP data management, functions for reading AP related data from files .
4	ReadWrite.h/.cpp	Structures for reading transportation network and activity program, custom classes for temporarily storing, reading, and writing data.
5	ConstructPVNet.h/.cpp	Constructing transportation network (car) using structs.
6	ConstructSNK.h/.cpp	Constructing multi-state supernetworks of an AP using structs.
7	GraphElements.h/.cpp	Custom classes designed for the graph elements (node and edge for transportation network and multi-state supernetwork).
8	Graph.h/.cpp	Custom classes designed for the graph (transportation network and multi-state supernetwork).
9	ConstructPVNSNK.h/.cpp	Constructing transportation and multi-state supernetwork using classes.
TBS path searching		
10	PathAndLabels.h/.cpp	Custom classes defined for paths in transportation network and multi-state supernetwork, and path searching using priority queue. Function declarations for TBS path searching functions.
11	ToolsTBS.cpp	Functions for TBS path searching.
STP construction, Visit Probability Simulation		
12	STPVPElements.h/.cpp	Custom classes and functions designed for manage STP results.
13	STPVPFuncs_Queue.h/.cpp	Functions for simulating visit probabilities.
14	SimulateVisitProbability_Queue.h/.cpp	Function for simulating visit probabilities for travel links and activity links at all selected time points and activity states (transitions).
15	MainVisitProbability.cpp	Function for simulating visit probability within STP of an AP (entry of the module (1)).
Accessibility Measurement Calculation		
16	AccessibilityMeasure.h/.cpp	Functions for calculating proposed accessibility measurement, function for calculating 3 traditional accessibility measurements (entry of module (3)).

17	MainAccessibilityMeasures.cpp	Function for calculating the proposed accessibility measurement (entry of the module (2)).
Read and Write files		
18	ReadWriteFile.h/.cpp	Functions for reading files from folders, and writing out files to folders.

C++ Code using guide

1. To run the Module (1):

   Steps:

   1) Set `bool CalculateVisitProbabilityNeeded = true`; in `MainFunctions.cpp`;
   2) Press `F5` or click the "`Local Windows Debugger`" button on the toolbar;
   3) The results will be written into the corresponding file folders (empty folders provided), which including:

      `"..\\..\\DataFiles\\NLNBATPsMSNSTPNodes\\"`: `NLNB_MSNSTPNodes_ATPID0.csv` (used in Module (2))

      `"..\\..\\DataFiles\\NLNBATPsEdgeVisitProbs\\TravelLinks\\"`: visit probabilities of travel links at different time points and activity states with name like: `NLNB_PVLinkID_TravelLinkVP_ATPID0_(time point)_TravelActState(activity state).csv` (used in Module (2))

      `"..\\..\\DataFiles\\NLNBATPsEdgeVisitProbs\\ActLinks\\"`: visit probabilities of activity links at different time points and activity state transitions with name like: `NLNB_FixedFlexActLocsVP_ATPID0_(time point)_(start activity state)To(end activity state).csv` (used in Module (2))

2. To run the Module (2):

   Steps:

   1) Set `bool CalculateAccessibilityMeasuresNeeded = true;` in `MainFunctions.cpp`;
   2) Press `F5` or click the "`Local Windows Debugger`" button on the toolbar;
   3) The code read files written in `"..\\..\\DataFiles\\NLNBATPsEdgeVisitProbs\\ActLinks\\"`. The results will be written into the corresponding file folders (empty folders provided), which including:

      `"..\\..\\DataFiles\\NLNBATPsAccessibilityMeasures\\ActLinks\\AMRes\\"`: *AVP* results for each time points and activity state transitions with name like: `AM_infos_NLNB_FixedFlexActLocs_ATPID0_(time point)_(start activity state)To(end activity state).csv` (used in Module (3))

      `"..\\..\\DataFiles\\NLNBATPsAccessibilityMeasures\\ActLinks\\AMResAllTStates\\"`: *AVP* summary result (single file)

3. To run the Module (3):

   Steps:

   1) Set `bool CalculateAMAFTNALMFTNeeded = true;` in `MainFunctions.cpp`;

2) Press `F5` or click the "`Local Windows Debugger`" button on the toolbar;
3) The code read files written in `"..\\..\\DataFiles\\NLNBATPsAccessibilityMeasures\\ActLinks\\AMRes\\`; the results will be written into the corresponding file folder (folder provided):
   `"..\\..\\DataFiles\\NLNBATPsAccessibilityMeasures\\ActLinks\\AMResAllTStates\\`: *AVP, NAL, AFT, MFT* summary result (single file)

Note also the following points:

1. To run the codes, one needs to make sure functions in the .cpp files read in the exact data files (or in the same form) as listed in Table 1.
2. If one do not run all modules at once, please set the `if` condition for the previous module to “`false`” in `MainFunctions.cpp` before running the current module.
3. If one wants to write out some specific outcomes, please activate the “write out” codes that provided in the .cpp files with self-defined path and file names.

Python Code for holding time density functions estimation

Code description:
The program was written in Python 3.10 and tested in PyCharm 2022.3.2. It should be compatible with various Python execution environments as long as the necessary interpreter and the required packages are installed. Only those important files for producing final results are explained. Necessary explanations for functions are written within the scripts.

The latent class models for holding time density functions are estimated based on the method of maximum likelihood estimation (MLE) using expectation–maximization (EM) algorithm.
`exponLatentClass_EM.py` is the code for estimating the parameters of the exponential latent class model, which are the parameters of exponential distribution for each latent class, and the coefficients in the membership function.
Similarly, `lognormalLatentClassMLE_EM.py` is the code for estimating the parameters of the lognormal latent class model.

Python Project Structure
```
/pythonCodes
    /MLE_EM (Project Folder)
        /.idea
        /DataFiles
            /data.xlsx
            / …
        /code1.py
        /…
```

Table 3 Holding time density functions estimation datasets.

	filename	contents
1	NonDaily_extraDura_3ageGroup_dummyCoding.xlsx	Individuals' socio-demographic characteristics and the extra shopping duration recorded in the daily mobility GPS trajectories.
2	Work_extraDura_3ageGroup_dummyCoding.xlsx	Individuals' socio-demographic characteristics and the extra working duration recorded in the daily mobility GPS trajectories.
3	ExtraTvlTime_3ageGroup_dummyCoding.xlsx	Individuals' socio-demographic characteristics and the extra travel time recorded in the daily mobility GPS trajectories.

Notes:

1. All the .xlsx data are using: dummy coding, 3 age group classification.

Table 4 Holding time density functions estimation python code files explanations.

	filename	descriptions
1	exponLatentClass_EM.py	Estimate exponential latent class model parameters and calculate associated statistical measures.
2	lognormalLatentClassMLE_EM.py	Estimate lognormal latent class model parameters and calculate associated statistical measures.

Python Code using guide

To run the `exponLatentClass_EM.py` or `lognormalLatentClassMLE_EM.py` in PyCharm:

Steps:

1) Set the `num_classes` to the value one wants to test (in line 770 or 832, respectively);
2) Run: `Shift + F10` or select "`Run`" from the right-click context menu on a file;
   Debug: `Shift + F9` or select "`Debug`" from the right-click context menu on a file;
3) Estimated parameter results and related statistical values will be displayed in the Console.

Note also the following points:

1. To run the codes, one needs to make sure functions read in the exact data files (or in the same form) as listed in Table 3.

==

The attached programs can be used in open science and application

Please contact the authors if the reader has any suggestions/remarks/questions.
